# GluN2B-NMDAR subunit contribution on synaptic plasticity: A phenomenological model for CA3-CA1 synapses

**DOI:** 10.3389/fnsyn.2023.1113957

**Published:** 2023-03-15

**Authors:** Justinas J. Dainauskas, Hélène Marie, Michele Migliore, Ausra Saudargiene

**Affiliations:** ^1^Laboratory of Biophysics and Bioinformatics, Neuroscience Institute, Lithuanian University of Health Sciences, Kaunas, Lithuania; ^2^Department of Informatics, Vytautas Magnus University, Kaunas, Lithuania; ^3^Université Côte d'Azur, Centre National de la Recherche Scientifique (CNRS) UMR 7275, Institut de Pharmacologie Moléculaire et Cellulaire (IPMC), Valbonne, France; ^4^Institute of Biophysics, National Research Council, Palermo, Italy

**Keywords:** synaptic plasticity, NMDA receptor, GluN2B-NMDA receptor subunit, CA1 pyramidal neuron, hippocampus

## Abstract

Synaptic plasticity is believed to be a key mechanism underlying learning and memory. We developed a phenomenological N-methyl-D-aspartate (NMDA) receptor-based voltage-dependent synaptic plasticity model for synaptic modifications at hippocampal CA3-CA1 synapses on a hippocampal CA1 pyramidal neuron. The model incorporates the GluN2A-NMDA and GluN2B-NMDA receptor subunit-based functions and accounts for the synaptic strength dependence on the postsynaptic NMDA receptor composition and functioning without explicitly modeling the NMDA receptor-mediated intracellular calcium, a local trigger of synaptic plasticity. We embedded the model into a two-compartmental model of a hippocampal CA1 pyramidal cell and validated it against experimental data of spike-timing-dependent synaptic plasticity (STDP), high and low-frequency stimulation. The developed model predicts altered learning rules in synapses formed on the apical dendrites of the detailed compartmental model of CA1 pyramidal neuron in the presence of the GluN2B-NMDA receptor hypofunction and can be used in hippocampal networks to model learning in health and disease.

## 1. Introduction

Long-term synaptic plasticity has been proposed to be the cellular substrate of learning and memory in the brain (Malenka and Nicoll, 1999; Malenka and Bear, 2004). In the hippocampal CA1 area, CA3 Schaffer collateral-CA1 pyramidal neuron synapses can undergo long-term potentiation (LTP) and long-term depression (LTD), triggered by high or low frequency presynaptic stimulation (Collingridge et al., 1983; Dudek and Bear, 1992; Mulkey and Malenka, 1992; Bliss and Collingridge, 1993; Goh and Manahan-Vaughan, 2013; Pousinha et al., 2017). Spike-timing-dependent synaptic plasticity (STDP) is a bidirectional form of synaptic modifications, induced by correlated pre- and postsynaptic neuronal activation, where precise timing of spikes is a major determinant of the direction and magnitude of synaptic modifications (Markram et al., 1997; Bi and Poo, 1998; Debanne et al., 1998; Feldman, 2012). The induction of LTP, LTD, and STDP in excitatory synapses at Schaffer collateral pathway requires activation of N-methyl-D-aspartate receptors (NMDARs) (Bliss and Collingridge, 1993, 2013; Collingridge and Bliss, 1995; Lüscher and Malenka, 2012; Volianskis et al., 2015). An NMDA-mediated rise in postsynaptic calcium triggers complex biochemical signaling pathways and translates into α-amino-3-hydroxy-5-methyl-4-isoxazole propionic acid receptors (AMPARs) insertion or removal (MacDermott et al., 1986; MacDonald et al., 2006; Lau et al., 2009) underlying LTP and LTD.

The function of NMDARs carries a profound effect on learning, memory, connectivity of neural networks in hippocampus, cognition, and psychiatric diseases (Buzsáki, 2002; Liu et al., 2004). NMDARs are composed of two GluN1 subunits and two GluN2 subunits, which may be of the GluN2A, GluN2B, GluN2C, and GluN2D type, and a pair of GluN3A and GluN3B subunits (Cull-Candy et al., 2001; Paoletti, 2011). NMDA GluN1/GluN2A channels exhibit faster kinetics than NMDA GluN1/GluN2B type channels (Cull-Candy et al., 2001). NMDA receptors in the hippocampus are composed mainly of GluN2A-NMDA and GluN2B-NMDA type subunits that are important for synaptic plasticity and normal memory functioning.

The GluN2B-NMDAR subunit plays a critical role in the induction of LTD and LTP. The deficits of GluN2B-NMDAR impaired LTP in hippocampal slices (Gardoni et al., 2009), and inhibition of this receptor subunit led to disruption or abolishment of synaptic plasticity in CA1 pyramidal neurons (Clayton et al., 2002; Berberich et al., 2005; Foster et al., 2010; Zamzow et al., 2013; France et al., 2017; Pousinha et al., 2017). Overexpression of the GluN2B-NMDAR subunit in the transgenic mice improved memory in cortex (Cui et al., 2011), while its blockade by the GluN2B-NMDAR subunit-specific antagonist, ifenprodil, disrupted fear memory (Zhao et al., 2005). Blocking GluN2B-NMDAR led the abolishment of LTP (Morishita et al., 2007; Andrade-Talavera et al., 2016; Pousinha et al., 2017) at hippocampal CA3-CA1 synapses.

GluN2B-NMDAR is strongly coupled with calcium-calmodulin-dependent protein kinase II (CaMKII), a key protein that induces downstream signaling cascades mediating LTP expression, learning, and memory. CaMKII leads to phosphorylation of synaptic proteins, increase in the number of active AMPARs or their single-channel conductance (Park et al., 2021; Yasuda et al., 2022). During LTP induction, *Ca*^2+^ influx through GluN2B-NMDAR directly activates CaMKII and leads to synapse strengthening (Shipton and Paulsen, 2014). Experimental data shows that disruption of GluN2B-NMDAR/CaMKII interactions downregulates CaMKII activation and autophosphorylation, prevents LTP in hippocampus, and impairs spatial learning in transgenic mice (Zhou et al., 2007). GluN2B-NMDA type of receptor is crucial for normal learning in hippocampus *in vivo* (Li et al., 2007). Moreover, GluN2B-NMDAR subunit is implicated in variety of psychiatric disorders like dementia, Alzheimer's disease (Liu et al., 2019), and Schizophrenia (Kocsis, 2012). GluN2B-NMDAR plays a critical role in synaptic plasticity and cognitive impairment in Alzheimer's disease (Parameshwaran et al., 2008; Pousinha et al., 2017, 2019).

In modeling studies of synaptic plasticity, the challenge is to integrate knowledge at molecular, synaptic, neuronal, and microcircuit levels, to understand the underlying LTP and LTD mechanisms in synapses and transfer the knowledge to large network simulations. Numerous computational studies of synaptic plasticity exist, and the models can be grouped into three main classes that employ phenomenological, optimal, and biophysical approach. Phenomenological models are abstract, and encode data and intuitions about synaptic plasticity taking into account spike timing (Gerstner et al., 1996; Kempter et al., 1999; Kistler and van Hemmen, 2000; Song and Abbott, 2000; Song et al., 2000). Optimal models use some optimality criterion to deduce the rules of synaptic modifications (Toyoizumi et al., 2005; Pfister et al., 2006). Biophysical models rely on biologically realistic descriptions of the electrophysiological and biochemical processes, usually are based on intracellular calcium dynamics, and involve detailed biochemical reactions to explain synaptic plasticity (Bhalla and Iyengar, 1999; Senn et al., 2001; Shouval et al., 2002; Badoual et al., 2006; Graupner and Brunel, 2007; Pi and Lisman, 2008; Clopath et al., 2010; Urbanczik and Senn, 2014; Migliore et al., 2015; Saudargiene et al., 2015; Sacramento et al., 2018; Ebner et al., 2019; Mäki-Marttunen et al., 2020; Chindemi et al., 2022).

In biophysical models a widely used approach is to investigate the molecular networks underlying synaptic plasticity such as CaMKII and protein phosphatase competition activated by NMDAR-mediated calcium (Graupner and Brunel, 2007; Pi and Lisman, 2008; Saudargiene et al., 2015). A well-known calcium control hypothesis states that low calcium levels in dendritic spine do not evoke any changes, intermediate calcium levels depress the synapse and high calcium transients potentiate the synapse (Shouval et al., 2002). Biophysical models, embedded into detailed compartmental models, account for the factors shaping synaptic plasticity—different membrane mechanisms of the dendritic tree, dendritic integration, morphological features, pattern of pre- and postsynaptic spiking (Poirazi and Papoutsi, 2020). The models include complex biochemical reactions of calcium induced kinase and phosphatase activation that underlie synaptic modifications (Bhalla and Iyengar, 1999; Graupner and Brunel, 2007; Saudargiene et al., 2015; Jędrzejewska-Szmek et al., 2017; Mäki-Marttunen et al., 2020), represent molecular cascades applying simplified functions, dependent on postsynaptic NMDAR-mediated intracellular calcium transients (Shouval et al., 2002; Graupner and Brunel, 2012; Standage et al., 2014; Chindemi et al., 2022), use formulation on the level of postsynaptic voltage (Clopath et al., 2010; Meissner-Bernard et al., 2020), utilize a kinetic model of synapse upregulation and downregulation mediated by NMDAR and based on the precise timing of pre and post spikes (Senn et al., 2001), or describe the weight change in a phenomenological way taking into account spike timing (Gerstner et al., 1996; Song and Abbott, 2000; Song et al., 2000). Phenomenological models are efficient, but lack biological realism; on the other hand, detailed models, sensitive to NMDAR functioning, are not easily applied in network simulations as they include many complex biochemical reactions, large parameter space, and are computationally expensive. The models that account for the NMDAR subunit properties and are suitable to analyze learning properties in networks are still lacking.

Different forms of LTP and LTD coexist that have different induction and expression mechanisms. In this study we focus on the GluN2B-NMDAR subunit effect on LTP induction in STDP, high and low frequency stimulation protocols. The aim of this work is to build a phenomenological NMDAR-based synaptic plasticity model that separates the influence of GluN2A-NMDAR and GluN2B-NMDAR subunits, and gain insight into the GluNR2B-NMDAR effect on synaptic modifications of hippocampal CA3-CA1 synapses. We modeled synaptic plasticity induced by a STDP protocol and high and low frequency stimulation, and explored the impact of GluN2B-NMDAR subunit properties on the modification of synaptic strength of the synapses spatially distributed on the apical dendrites of CA1 pyramidal neuron. The novelty of the work is the approach to include the influence of the specific NMDA receptor subunits on synaptic plasticity as the separate mediators of LTP and LTD. We assume that LTP is mainly mediated by GluN2B-NMDAR (Morishita et al., 2007; Andrade-Talavera et al., 2016; Pousinha et al., 2017), and LTD is triggered by other mechanisms, possibly including GluN2A-NMDAR. Experimental studies showed that GluN2A-NMDAR blockade prevented LTD induction in the CA1 region of hippocampal slices (Bartlett et al., 2007; Li et al., 2007). The study of Morishita et al. (2007) also suggested that GluN2A-NMDAR might be responsible for LTD as the application of the GluN2B-NMDAR antagonist ifenprodil did not prevent the induction of LTD by low frequency stimulation.

We analyzed the effect of GluN2B-NMDAR on the properties of learning at the synapses of hippocampal CA1 pyramidal neuron. The modeling results provide insights into the learning rules of hippocampal CA1 pyramidal neuron synapses in healthy and GluN2B-NMDAR hypofunction conditions.

## 2. Methods

We developed a model of synaptic modifications based on the well-established phenomenological models (Clopath et al., 2010; Meissner-Bernard et al., 2020) and integrated the separated influence of postsynaptic NMDAR subunits GluN2A-NMDAR and GluN2B-NMDAR to account for the crucial effect of GluN2B-NMDAR in hippocampal synaptic plasticity. We utilized two computational models of CA1 pyramidal neuron: a modified two-compartmental Pinsky-Rinzel model (Pinsky and Rinzel, 1994; Ferguson and Campbell, 2009) to validate the extended synaptic plasticity model, and a multicompartmental model (Migliore et al., 2018) to study the influence of GluN2B-NMDAR properties on synaptic strength modifications at a cluster of CA3-CA1 synapses distributed randomly onto apical dendrites of CA1 neuron.

### 2.1. NMDAR-based voltage-dependent synaptic plasticity model

We extended a voltage-based model of synaptic plasticity (Clopath et al., 2010; Meissner-Bernard et al., 2020) by including the effect of postsynaptic NMDAR subunits GluN2A-NMDAR and GluN2B-NMDAR. The instantaneous weight change $\frac{d}{dt}\left(t\right)w$ consists of two additive NMDAR-dependent LTD and LTP contributions, $\frac{d}{dt}w_{LTP}\left(t\right)$ and $\frac{d}{dt}w_{LTD}\left(t\right)$, following Clopath et al. (2010) and Meissner-Bernard et al. (2020):


(1) $$\begin{array}{l} \begin{array}{c} \frac{d}{dt}w\left(t\right)=\frac{d}{dt}w_{LTP}\left(t\right)\left(w_{max}-w\left(t\right)\right)-\frac{d}{dt}w_{LTD}\left(t\right)\left(w\left(t\right)-w_{min}\right), \end{array} \end{array}$$

where *w*_*max*_ and *w*_*min*_ set the limits for synaptic weight *w*. The LTP component $\frac{d}{dt}w_{LTP}\left(t\right)$ is expressed as the product of the NMDAR-dependent function ϕ_*NMD*_*A*__+__(*t*) and a low-filtered membrane potential ${\bar{V}}_{+}\left(t\right)$:


(2) $$\begin{array}{l} \begin{array}{c} \frac{d}{dt}w_{LTP}\left(t\right)=A_{+}\;\phi_{NMDA_{+}}\left(t\right)\;{\bar{V}}_{+}\left(t\right), \end{array} \end{array}$$

where *A*_+_ is the LTP amplitude parameter. Similarly, the LTD component $\frac{d}{dt}w_{LTD}\left(t\right)$ is proportional to the product of the NMDAR-dependent function ϕ_*NMD*_*A*__−__(*t*) and a low-filtered membrane potential ${\bar{V}}_{-}\left(t\right)$:


(3) $$\begin{array}{l} \begin{array}{c} \frac{d}{dt}w_{LTD}\left(t\right)=A_{-}\;\phi_{NMDA_{-}}\left(t\right)\;{\bar{V}}_{-}\left(t\right)\;\bar{X}\left(t\right), \end{array} \end{array}$$

where $\bar{X}\left(t\right)$ is a presynaptic activity variable, and *A*_−_ is the LTD amplitude parameter.

The contribution of the postsynaptic GluN2A-NMDAR and GluN2B-NMDAR gated channel is captured by the newly introduced Hill function ϕ_*NMD*_*A*__[*]__(*t*), here [*] indicates the LTP and LTD components ([*] is [+] for LTP and [−] for LTD):


(4) $$\begin{array}{l} \begin{array}{c} \phi_{NMDA_{\left[*\right]}}\left(t\right)=\frac{1}{1+{\left(\frac{K_{a\left[*\right]}}{{\bar{g}}_{NMDA_{\left[*\right]}}\left(t\right)}\right)}^{n_{\left[*\right]}}}-\theta_{\phi_{\left[*\right]}}, \end{array} \end{array}$$

where ${\bar{g}}_{NMDA_{\left[*\right]}}\left(t\right)$ is the filtered NMDAR conductance, *K*_*a*[*]_ is a value of ${\bar{g}}_{NMDA_{\left[*\right]}}\left(t\right)$, producing half activation of ϕ_*NMD*_*A*__[*]__(*t*), *n*_[*]_ is the Hill coefficient, and θ_ϕ_[*]__ is a threshold of ϕ_*NMD*_*A*__[*]__(*t*) for LTP and LTD induction. Values of *n*_[*]_, *K*_*a*[*]_, and θ_ϕ_[*]__ differ for the LTD and LTP contributions. Function ϕ_*NMD*_*A*__+__(*t*) governs LTP induction and is caused by the filtered NMDAR conductance ${\bar{g}}_{NMDA_{+}}\left(t\right)$. Function ϕ_*NMD*_*A*__−__(*t*) accounts for LTD, and is triggered by the filtered NMDAR conductance ${\bar{g}}_{NMDA_{-}}\left(t\right)$.

The moving threshold function θ_ϕ_[*]__ lowers ϕ_*NMD*_*A*__[*]__(*t*) activity and implements competition between LTP and LTD:


(5) $$\begin{array}{l} \begin{array}{c} \tau_{\theta_{\phi_{\left[*\right]}}}\frac{d}{dt}\theta_{\phi_{\left[*\right]}}\left(t\right)=-\theta_{\phi_{\left[*\right]}}\left(t\right)+b_{\theta_{\phi_{\left[*\right]}}}\;\phi_{NMDA_{\left[*\right]}}\left(t\right), \end{array} \end{array}$$

where τ_θ__ϕ__[*]___ is a time constant and *b*_θ__ϕ__[*]___ is a scaling coefficient, and [*] denotes [−] for LTP and [+] for LTP components.

The moving threshold θ_ϕ_−_(*t*)_ is increasing, if ϕ_*NMD*_*A*__+__(*t*) is strongly activated and LTP is induced, thus vetoing LTD. Threshold θ_ϕ_+__(*t*) may also increase if ϕ_*NMD*_*A*__−__(*t*) accumulates, and leads to LTD.

The filtered NMDAR-dependent variables ${\bar{g}}_{NMDA_{\left[*\right]}}\left(t\right)$ for LTP and LTD components are described:


(6) $$\begin{array}{l} \tau_{NMDA_{\left[*\right]}}\frac{d}{dt}{\bar{g}}_{NMDA_{\left[*\right]}}\left(t\right)=-{\bar{g}}_{NMDA_{\left[*\right]}}\left(t\right)+g_{NMDA}\left(t\right), \end{array}$$

where τ_*NMD*_*A*__[*]__ is a time constant, and *g*_*NMD*_*A*__[*]__(*t*) is a conductance of postsynaptic NMDAR that incorporates both GluN2A-NMDAR and GluNB-NMDAR subunits with a different weighting coefficient *k*_2*B*[*]_ for LTP and LTD components:


(7) $$\begin{array}{l} \begin{array}{c} g_{NMDA_{\left[*\right]}}\left(t\right)=k_{2B\left[*\right]}\;g_{NMDA_{GluN2B}}\left(t\right)+\left(1-k_{2B\left[*\right]}\right)\;g_{NMDA_{GluN2A}}\left(t\right) \end{array} \end{array}$$

Following Morishita et al. (2007), Andrade-Talavera et al. (2016), and Pousinha et al. (2017), we assume that LTP is mainly governed by GluN2B-NMDAR subunit, and LTD is mediated by GluN2A-NMDAR (or other) subunit. We set the coefficient of GluN2B-NMDAR effect on LTP *k*_2*B*+_ = 0.8, and coefficient of GluN2B-NMDAR effect on LTD *k*_2*B*−_ = 0.2.

GluN2B-NMDAR subunit has a slower inactivation time than GluN2A-NMDAR subunit. Kinetic parameters of forward and backward binding rates are adjusted (Cull-Candy et al., 2001).

Synaptic conductances of GluN2A-NMDAR and GluNB-NMDAR subunits are modeled following Destexhe et al. (1994):


(8) $$\begin{array}{l} \begin{array}{c} g_{NMDA_{\left[GluN2†\right]}}=f_{Mg}\left(R_{on_{\left[GluN2†\right]}}-R_{off_{\left[GluN2†\right]}}\right){ĝ}_{NMDA_{\left[GluN2†\right]}}, \end{array} \end{array}$$

where [*GluN*2†] denotes two types of NMDAR GluN2 subunits, GluN2A-NMDAR and GluN2B-NMDAR, *R*_*o*_*n*__[*GluN*2†]__ and *R*_*of*_*f*__[*GluN*2†]__ are the fraction of open and closed GluN2A-NMDAR and GluNB-NMDAR, ĝ_*NMD*_*A*__[*GluN*2†]__ is the maximal GluN2A-NMDAR and GluN2B-NMDAR conductances, and $f_{\left[Mg^{2+},V\left(t\right)\right]}$ is a NMDAR gating function, dependent of extracellular magnesium concentration [*Mg*^2+^] and local membrane potential *V*(*t*):


(9) $$\begin{array}{l} \begin{array}{c} f_{\left[Mg^{2+},V\left(t\right)\right]}=\frac{1}{\left(1+e^{-0.062V\left(t\right)}\right)\left(\left[Mg^{2+}\right]/3.57\right)}. \end{array} \end{array}$$

*R*_*o*_*n*__[*GluN*2†]__, *R*_*of*_*f*__[*GluN*2†]__, and *R*_*in*_*f*__[*GluN*2†]__ of GluN2A-NMDAR and GluNB-NMDAR are equal:


(10) $$\begin{array}{l} \begin{array}{c} \tau_{\left[GluN2†\right]}\frac{d}{dt}R_{on_{\left[GluN2†\right]}}=R_{inf_{\left[GluN2†\right]}}-R_{on_{\left[GluN2†\right]}},\\ \frac{d}{dt}R_{off_{\left[GluN2†\right]}}=-\beta_{\left[GluN2†\right]}R_{off_{\left[GluN2†\right]}}, \end{array} \end{array}$$

and


(11) $$\begin{array}{l} \begin{array}{c} R_{inf_{\left[GluN2†\right]}}=\frac{\alpha_{\left[GluN2†\right]}}{\alpha_{\left[GluN2†\right]}+\beta_{\left[GluN2†\right]}}, \end{array} \end{array}$$

where α_[*GluN*2†]_ and β_[*GluN*2†]_ are forward and backward binding rates of GluN2A-NMDAR and GluN2B-NMDAR, adjusted following (Cull-Candy et al., 2001).

Time constant τ_[*GluN*2†]_ is defined:


(12) $$\begin{array}{l} \begin{array}{c} \tau_{\left[GluN2†\right]}=\frac{1}{\alpha_{\left[GluN2†\right]}+\beta_{\left[GluN2†\right]}}. \end{array} \end{array}$$

Variables ${\bar{V}}_{-}\left(t\right)$ and ${\bar{V}}_{+}\left(t\right)$ are the functions of the filtered membrane potential *V*(*t*) at the synapse location, and contribute to the LTD and LTP components:


(13) $$\begin{array}{l} \begin{array}{c} \tau_{\left[*\right]}\frac{d}{dt}{\bar{V}}_{\left[*\right]}\left(t\right)=-{\bar{V}}_{\left[*\right]}\left(t\right)+{\left[V\left(t\right)-\theta_{\left[*\right]}\right]}_{+}, \end{array} \end{array}$$

where τ_[*]_ is a time constant and θ_[*]_ is a threshold for the LTP and LTD components.

A presynaptic activity variable $\bar{X}$ in Equation (3) is calculated as a low pass filter of the presynaptic spike train Σ_*i*_δ(*t−t*_*i*_) with time constant τ_δ_ using $\tau_{\delta }\frac{d}{dt}\bar{X}\left(t\right)=-\bar{X}\left(t\right)+\Sigma_{i}\delta \left(t-t_{i}\right)$.

**Figure 1:**
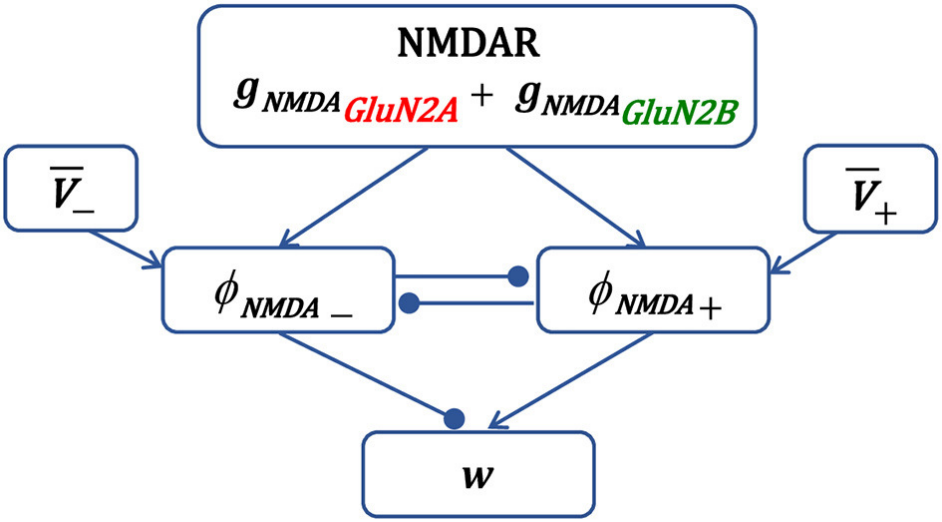
Schematic diagram of the main components of synaptic plasticity model. Presynaptic action potential activates NMDAR, induces the NMDAR conductance, composed of GluN2A-NMDAR and GluN2B-NMDAR subunits *g*_*NMD*_*A*__*GluN*2*A*__ and *g*_*NMD*_*A*__*GluN*2*B*__, and triggers variables ϕ_*NMD*_*A*__+__ and ϕ_*NMD*_*A*__−__ that account for the LTP and LTD contribution, respectively. The LTP variable ϕ_*NMD*_*A*__+__, once activated, inhibits the LTD variable ϕ_*NMD*_*A*__−__ to prevent LTD, and vice versa. Postsynaptic local membrane potential *V* is low-pass filtered, and the resulting LTP and LTD variables ${\bar{V}}_{+}$ and ${\bar{V}}_{-}$ are multiplied by the corresponding NMDAR-dependent variables ϕ_*NMD*_*A*__+__ and ϕ_*NMD*_*A*__−__ to form the LTP and LTD components of the weight *w*.

Schematic representation of synaptic plasticity model is shown in Figure 1. The presynaptic activity triggers NMDAR synaptic conductance *g*_*NMDA*_(*t*), composed of GluN2A-NMDAR and GluN2B-NMDAR subunits, and induces variables ϕ_*NMD*_*A*__+__(*t*) and ϕ_*NMD*_*A*__−__(*t*). Once activated, the LTP variable ϕ_*NMD*_*A*__+__(*t*) inhibits the LTD variable ϕ_*NMD*_*A*__−__(*t*) preventing LTD induction, and vice versa—ϕ_*NMD*_*A*__−__(*t*) may reduce the activity of ϕ_*NMD*_*A*__+__(*t*). Postsynaptic activity is captured by a local membrane potential *V*(*t*) that is thresholded using the thresholds θ_+_ and θ_−_, and low-pass filtered resulting in ${\bar{V}}_{+}\left(t\right)$ and ${\bar{V}}_{-}\left(t\right)$. The LTP component $\frac{d}{dt}w_{LTP}\left(t\right)$ is obtained by multiplying *V*_+_(*t*) and ϕ_*NMD*_*A*__+__(*t*), and the LTD component $\frac{d}{dt}w_{LTD}\left(t\right)$ is a product of *V*_−_(*t*), ϕ_*NMD*_*A*__−__(*t*), and $\bar{X}\left(t\right)$. The weight change of the AMPAR strength *w* is composed of the scaled LTP and LTD parts $\frac{d}{dt}w_{LTP}\left(t\right)$ and $\frac{d}{dt}w_{LTD}\left(t\right)$.

The novelty of the model is that it captures the separate specific influence of GluN2A-NMDAR and GluN2B-NMDAR subunits on LTP and LTD induction using the NMDAR subunit-dependent activation functions ϕ_*NMD*_*A*__−__(*t*) and ϕ_*NMD*_*A*__+__(*t*) (Equation 4).

The GluN2A-NMDAR and GluN2B-NMDAR subunit-dependent functions (Equations 4, 6, 7) shape the LTP and LTD components (Equations 2, 3). The filtered NMDAR synaptic conductance-dependent variables ${\bar{g}}_{NMDA_{\left[*\right]}}\left(t\right)$ (Equation 6) can be interpreted as the intracellular *Ca*^2+^ concentration, and functions ϕ_*NMD*_*A*__[*]__(*t*) (Equation 4) reflect the activation of intracellular *Ca*^2+^-triggered second messenger cascades underlying synaptic plasticity induction. Specifically, ϕ_*NMD*_*A*__+__(*t*) may represent phosphorylation of CaMKII, leading to LTP, and ϕ_*NMD*_*A*__−__(*t*) may indicate dephosphorylation of protein phosphatase 2B (PP2B, calcineurin), responsible for LTD. The description of the signaling pathways is simplified and implemented in a phenomenological manner using the NMDAR-dependent functions. The model does not require the estimation of the intracellular calcium concentration at a postsynaptic site and relies on the NMDAR properties and local membrane potential.

The parameters of the synaptic plasticity model are given in Table 1. The parameters of the GluN2A-NMDAR and GluN2B-NMDAR synaptic conductances are presented in Table 2.

**Table 1:** Parameters of synaptic plasticity model.

**Parameter**	**Value**	**Unit**	**Description**
*A*_+_	1 (8 x 10^−4^); (9 x 10^−2^)	1/(*mVms*)	Amplitude of LTP
*A*_−_	1 x 10^2^ (2 x 10^4^); (9 x 10^−1^)	1/(*mVms*)	Amplitude of LTD
*K*_*a*+_	11 x 10^−5^ (5 x 10^−2^); (7 x 10^−3^)	μ*S*/*cm*^2^	Value of the filtered ${\bar{g}}_{NMDA_{+}}\left(t\right)$ producing half occupation of ϕ_*NMD*_*A*__+__(*t*) for LTP component
*K*_*a*−_	9 x 10^−5^ (2 x 10^−2^); (4 x 10^−3^)	μ*S*/*cm*^2^	Value of the filtered ${\bar{g}}_{NMDA_{-}}$ producing half occupation of ϕ_*NMD*_*A*__−__(*t*) for LTD component
τ_*NMD*_*A*__+__	20	*ms*	Time constant of the filtered ${\bar{g}}_{NMDA_{+}}\left(t\right)$ for LTP component
τ_*NMD*_*A*__−__	1, 000	*ms*	Time constant of the filtered ${\bar{g}}_{NMDA_{-}}\left(t\right)$ for LTD component
*n*_+_	4	1	Hill coefficient of ϕ_*NMD*_*A*__+__(*t*) for LTP component
*n*_−_	2	1	Hill coefficient of ϕ_*NMD*_*A*__−__(*t*) for LTD component
τ_θ_ϕ+__	100	*ms*	Time constant of the moving threshold θ_*H*_(*t*) for LTP component
τ_θ_ϕ−__	100	*ms*	Time constant of the moving threshold θ_*H*_(*t*) for LTD component
*b*_θ_ϕ+__	10^1^; (10^2^); (10^−1^)	1	Coefficient of the moving threshold θ_*H*_(*t*) for LTP component
*b*_θ_ϕ−__	10^3^; (10^2^); (10^−1^)	1	Coefficient of the moving threshold θ_*H*_(*t*) for LTD component
θ_+_	−65;(−67);(−67)	*mV*	Threshold of *V*(*t*) for LTP component
θ_−_	−67	*mV*	Threshold of *V*(*t*) for LTD component
τ_+_	10	*ms*	Time constant of the filtered ${\bar{V}}_{+}\left(t\right)$ for LTP component
τ_−_	10	*ms*	Time constant of the filtered ${\bar{V}}_{-}\left(t\right)$ for LTD component
τ_δ_	15	*ms*	Dirac delta trace time constant
*w*_*min*_	0.4 (0.2)	1	Minimum weight value
*w*_*max*_	2.0 (2.5)	1	Maximum weight value

Parameter values are presented for two-compartmental model and multicompartmental model (in separate parentheses for Figures 6, 7A, and for Figure 7B, if different) of CA1 pyramidal neuron.

**Table 2:** Parameters of NMDAR synapse.

**Parameter**	**Value**	**Unit**	**Description**	**References**
**GluN2A-NMDAR and GluN2B-NMDAR**				
α_*GluN*2*A*_	0.5	/*ms*	Forward binding rate of GluNR2A NMDAR	Fitted (Cull-Candy et al., 2001)
β_*GluN*2*A*_	0.024	/*ms*	Backward binding rate of GluNR2A NMDAR	Fitted (Cull-Candy et al., 2001)
α_*GluN*2*B*_	0.1	/*ms*	Forward binding rate of GluNR2B NMDAR	Fitted (Cull-Candy et al., 2001)
β_*GluN*2*B*_	0.0075	/*ms*	Backward binding rate of GluNR2B NMDAR	Fitted (Cull-Candy et al., 2001)
ĝ_*NMD*_*A*__*GluN*2*A*__	1 x 10^−2^ (5.1 x 10^−5^)	*nS*	Maximal GluNR2A NMDAR conductance	Adjusted
ĝ_*NMD*_*A*__*GluN*2*B*__	1 x 10^−2^ (5.1 x 10^−5^)	*nS*	Maximal GluNR2B NMDAR conductance	Adjusted
[*Mg*^2+^]	1	*mM*	Extracellular magnesium concentration	Destexhe et al., 1994

Parameter values are presented for synapses in two-compartmental model and multicompartmental model (in parentheses, if different) of CA1 pyramidal neuron.

### 2.2. Computational models of CA1 pyramidal neuron

We employed two computational models of CA1 pyramidal neuron: a modified two-compartmental Pinsky-Rinzel model for synaptic plasticity model validation (Pinsky and Rinzel, 1994; Ferguson and Campbell, 2009) and a compartmental detailed model (Migliore et al., 2018) for analysis of GluN2B-NMDAR-dependent synaptic plasticity properties at CA3-CA1 synapses distributed on the apical dendrites of CA1 pyramidal neuron in the stratum radiatum (SR) region.

#### 2.2.1. Two-compartmental model of CA1 pyramidal neuron

A two-compartmental Pinsky-Rinzel model consisted of a somatic and dendritic compartments connected by the coupling conductance (Pinsky and Rinzel, 1994; Ferguson and Campbell, 2009). The somatic compartment had five ionic current channels: inward *Na*^+^ current *I*_*Na,s*_, outward delayed rectifier *K*^+^ current *I*_*KDR,s*_, inward *Ca*^2+^ current *I*_*Ca,s*_, outward short-duration voltage and *Ca*^2+^- dependent *K*^+^ current *I*_*KCa,s*_, and outward long-duration *Ca*^2+^-dependent after hyperpolarization (AHP) *K*^+^ current *I*_*KAHP,s*_. The dendritic compartment had three ionic current channels: inward *Ca*^2+^ current *I*_*Ca,d*_, outward short-duration voltage and *Ca*^2+^-dependent *K*^+^ current *I*_*KCa,d*_, outward long-duration *Ca*^2+^-dependent AHP potassium current *I*_*KAHP,d*_. Both compartments had leak current *I*_*leak,s*_, *I*_*leak,d*_. A single synapse containing AMPAR and GluN2A-NMDAR/GluN2B-NMDAR was formed on the dendritic compartment. The two-compartment model and synaptic currents are described in Supplementary material. The partial blockade of GluN2B-NMDAR-gated channel was simulated by reducing the conductance *g*_*NMD*_*A*__*GluN*2*B*__ (Equation 7).

#### 2.2.2. Multicompartmental model of CA1 pyramidal neuron

A multicompartmental model of a CA1 pyramidal cell oh140807_A0_idA (Migliore et al., 2018) consisting of 175 compartments was used, and it included 11 ionic current channels and a leak current. The ionic currents were the following: inward *Na*^+^ current *I*_*Na*_; four types of *K*^+^ currents: outward delayed rectifier *K*^+^ current *I*_*KDR*_, transient A-type *K*^+^ current *I*_*KA*_, currents *I*_*KM*_, *I*_*KD*_; three types of inward *Ca*^2+^ currents: N-type current *I*_*CaN*_, L-type current *I*_*CaL*_, T-type current *I*_*CaT*_; two types of *Ca*^2+^-dependent *K*^+^ currents: outward short-duration voltage and *Ca*^2+^-dependent *K*^+^ current *I*_*KCa*_ and *I*_*Cagk*_ current; and the non-specific *I*_*h*_ current. Ionic channels were uniformly distributed in all dendritic compartments except *I*_*KA*_ and *I*_*h*_, which increased with distance from the soma. Channels were described using a conventional Hodgkin-Huxley formalism, and peak conductances of each channel were optimized for soma, axon, basal, and apical dendrite compartments and validated against experimental data. Intracellular calcium concentration was described by a simple *Ca*^2+^ extrusion mechanism with a single exponential decay. The multicompartmental model and synaptic currents are described in Migliore et al. (2018).

A cluster of 50 AMPARs and GluN2A/GluN2B-NMDARs containing synapses, distributed randomly on the apical dendrites of the neuron in the SR region at 140 μ*m* from the soma with a synaptic density of 0.8 synapses/μ*m* of dendrite (Gasparini et al., 2004; Bezaire et al., 2016) was formed to model synaptic modifications.

The ratio of AMPAR/NMDAR gated channel currents was replicated using the experimental protocol used in (Pousinha et al., 2017). The voltage was clamped at -65 mV for AMPAR gated channel current estimation, and at +40 mV for NMDAR gated channel assessment. The peak AMPAR current was compared with the NMDAR current 60 ms after the onset of stimulus. The maximal conductances of the AMPAR and NMDAR-gated channels were set to ensure this ratio to be equal to 4 as in Pousinha et al. (2017). The partial blockade of GluN2B-NMDAR-gated channel was simulated by lowering the conductance *g*_*NMD*_*A*__*GluN*2*B*__ (Equation 7).

### 2.3. Stimulation protocols for synaptic plasticity induction at CA3-CA1 synapses

Synapses were stimulated using the activation patterns applied in the following electrophysiological studies of synaptic plasticity:

- STDP induction protocol (Wittenberg and Wang, 2006; Inglebert et al., 2020). Presynaptic input was paired with a doublet of postsynaptic action potentials 60 times at 5 Hz; 5 times at 5 Hz frequency; and 30 times at 1 Hz. Temporal difference Δ*T* was measured between pre- and a second postsynaptic spike. In addition, a presynaptic spike was paired with a single postsynaptic action potential 60 times at 5 Hz. Temporal difference Δ*T* was measured between a pre- and a postsynaptic spike (Wittenberg and Wang, 2006). Pairing frequency was increased from 1 to 50 Hz for spike pairs with a temporal difference Δ*T* = 10 ms between a pre- and a single postsynaptic spike. The number of postsynaptic spikes was varied from one to four with a temporal difference Δ*T* = 10 ms between pre- and the first postsynaptic spike (Inglebert et al., 2020). The spike pairings were repeated 30 times. Somatic action potential was induced by current pulse injection into the soma.
- Frequency-dependent synaptic plasticity induction protocol (Pousinha et al., 2017). Presynaptic input was stimulated at 100 Hz for 1 s (LTP protocol) or at 1 Hz for 100 s (LTD protocol). To estimate the change in the excitatory postsynaptic potential (EPSP), a presynaptic stimulus was delivered before and after the conditioning stimulation, and the resulting ratio between the maximal values of the resulting EPSPs was calculated.

Simulations were performed in the Python and NEURON simulation environment (version 8.0.0) (Hines and Carnevale, 1997). All model files in Python are available for public download under the ModelDB section of the Senselab database, accession number 267680 (https://senselab.med.yale.edu/ModelDB/).

## 3. Results

### 3.1. Validation of synaptic plasticity model

The developed synaptic plasticity model was validated against experimental data (Wittenberg and Wang, 2006; Pousinha et al., 2017; Inglebert et al., 2020).

**Figure 2:**
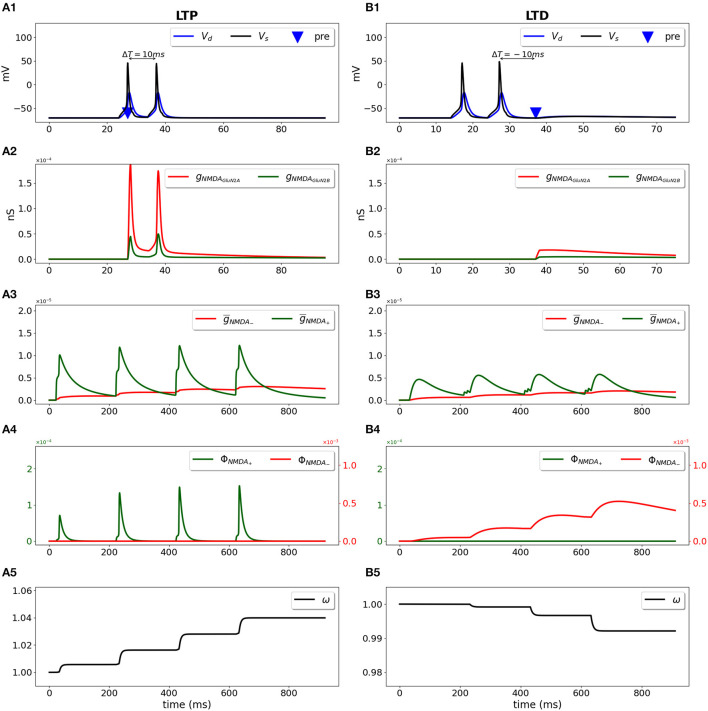
Synaptic weight change for the STDP induction protocol using a two-compartment model of CA1 pyramidal neuron. Presynaptic input was paired with a doublet of postsynaptic action potentials with the temporal difference between pre- and a second postsynaptic activity Δ*T* = 10 ms **(A1–A5)** and Δ*T* = -10 ms **(B1–B5)**. **(A1, B1)** Membrane potential in soma *V*_*s*_ (black line) and dendrite *V*_*d*_ (blue line); presynaptic input is indicated by a blue triangle; **(A2, B2)** GluN2A-NMDAR and GluN2B-NMDAR conductances *g*_*NMD*_*A*__*GluN*2*A*__ (red line) and *g*_*NMD*_*A*__*GluN*2*B*__ (green line); **(A3, B3)** filtered NMDAR-dependent variables ${\bar{g}}_{NMDA_{-}}$ (red) and ${\bar{g}}_{NMDA_{+}}$ (green) for LTD and LTP; **(A4, B4)** NMDAR-dependent functions ϕ_*NMD*_*A*__−__ (red line) and ϕ_*NMD*_*A*__+__ (green line) for the LTD and LTP components; **(A5, B5)** synaptic weight *w*.

We used a two-compartment neuron model with a single synapse on its dendrite, and applied the STDP induction protocol by pairing presynaptic activity with a doublet of postsynaptic action potentials (Wittenberg and Wang, 2006) (Figure 2). For pre-post stimulation protocol and Δ*T* = 10 ms, the presynaptic activation precedes a second action potential (Figure 2A1, blue triangle and black line, respectively) and results in opening of GluN2A-NMDAR *g*_*NMD*_*A*__*GluN*2*A*__ (red line) and GluN2B-NMDAR *g*_*NMD*_*A*__*GluN*2*B*__ (green line) (Figure 2A2); filtered NMDAR-dependent variables ${\bar{g}}_{NMDA_{-}}$ (red) and ${\bar{g}}_{NMDA_{+}}$ (green) for LTD and LTP induction (Figure 2A3) favor activation of the LTP function ϕ_*NMD*_*A*__+__ (Figure 2A4, green line). The product of ϕ_*NMD*_*A*__+__ and ${\bar{V}}_{+}$ forms $\frac{d}{dt}w_{LTP}\left(t\right)$ and leads to the increased weight *w* (Figure 2A5). For post-pre stimulation protocol and Δ*T* = −10 ms, the presynaptic activation follows a second somatic action potential (Figure 2B1, blue triangle and black line, respectively), NMDAR activation is weaker (Figure 2B2), failing to sufficiently activate ϕ_*NMD*_*A*__+__, but strong enough to induce ϕ_*NMD*_*A*__−__ (Figure 2B4, green and red lines, respectively). Functions ϕ_*NMD*_*A*__−__, ${\bar{V}}_{-}$, and $\bar{X}\left(t\right)$ combine into $\frac{d}{dt}w_{LTD}\left(t\right)$ and result in the decreased weight *w* (Figure 2B5).

**Figure 3:**
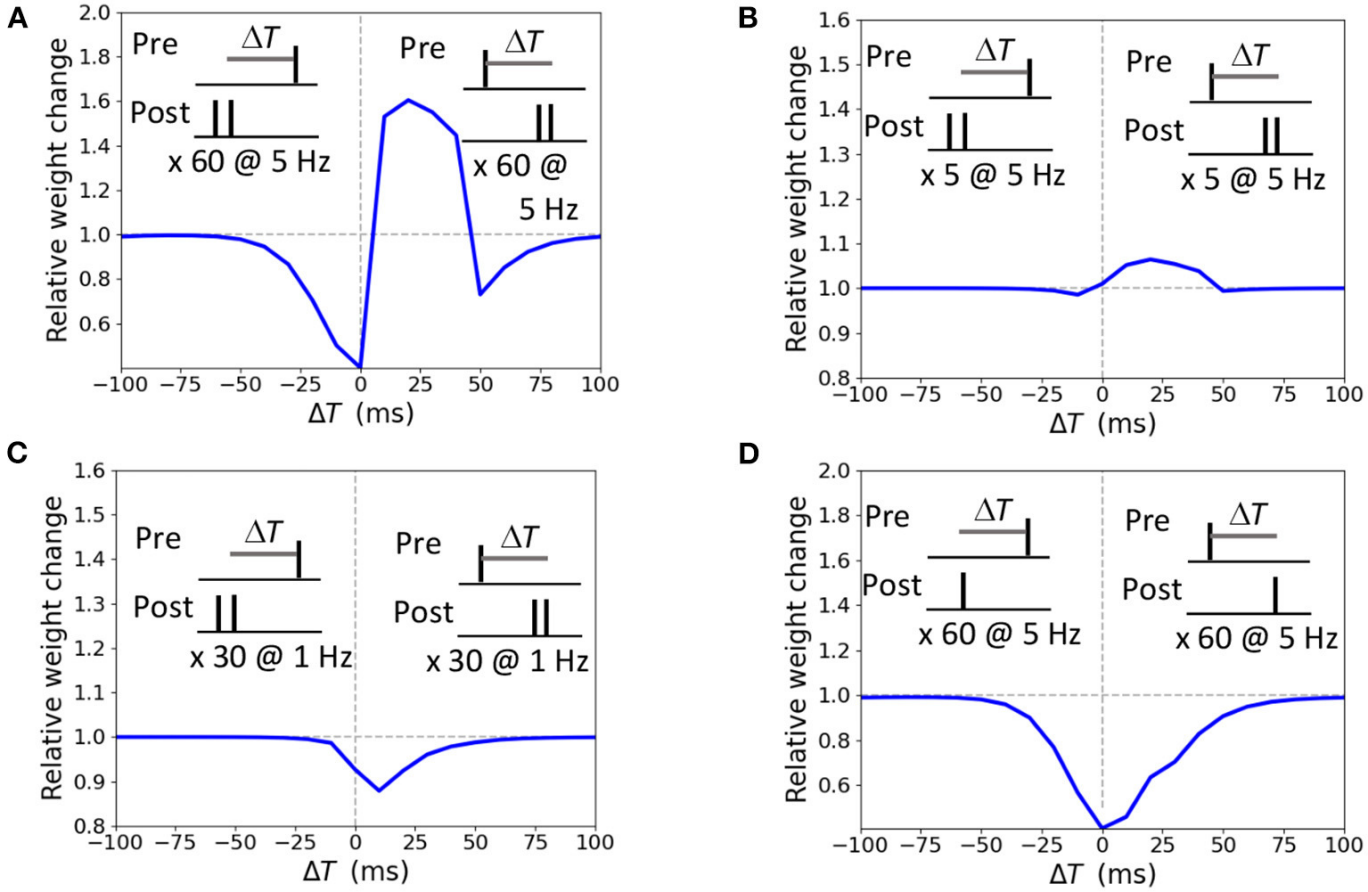
Synaptic modifications induced by pairing a presynaptic action potential with a doublet **(A–C)** or a single postsynaptic action potential **(D)**. Temporal difference Δ*T* is measured between the presynaptic and a second postsynaptic action potential **(A–C)** or a single postsynaptic action potential **(D)**. **(A)** 60 pairings at 5 Hz lead to LTP and two LTD windows; **(B)** 5 pairings at 5 Hz induce LTP; **(C)** 30 pairings at 1 Hz triggers LTD; **(D)** 60 pairings at 5 Hz results in LTD.

The STDP curve of the weight change Δ*w* induced by pairing the presynaptic input with a doublet of postsynaptic action potential 60 times at 5 Hz frequency with temporal difference Δ*T* ∈ [-100; 100 ms] is presented in Figure 3A. For the positive Δ*T* window from 0 ms up to 40 ms a synapse undergoes LTP, and LTD is obtained for anti-causal pairings and causal pairings within the Δ*T* interval [40; 100 ms]. Shorter stimulation of five pairings at 5 Hz results in a potentiation-only plasticity rule (Figure 3B) as the duration is not sufficient for the accumulation of the LTD mechanisms activity (ϕ_*NMD*_*A*__−__ in our model). Pairings at low frequency of 1 Hz results in LTD only (Figure 3C). A single postsynaptic action potential paired with a presynaptic action potential 60 times at 5 Hz evokes LTD, as the activation of LTP variable is too weak (ϕ_*NMD*_*A*__+__ in our model, Figure 3D). The modeled STDP weight modifications replicate the experimental data (Wittenberg and Wang, 2006).

**Figure 4:**
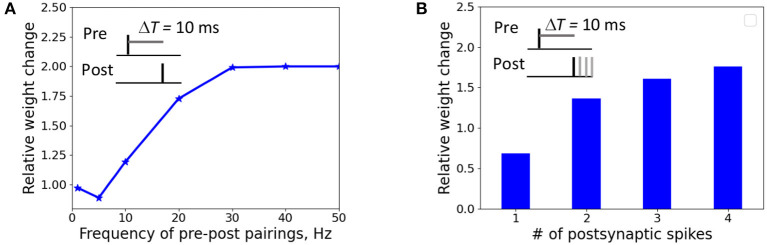
Weight change dependence on frequency of postsynaptic pairings **(A)** and a number of postsynaptic spikes **(B)**. **(A)** A presynaptic action potential was paired with a single postsynaptic action potential 20 times at Δ*T* = 10 ms. **(A)** A presynaptic action potential was paired with a one to four postsynaptic action potentials 30 times at 5 Hz at Δ*T* = 10 ms.

We investigated the weight change dependence on frequency of postsynaptic pairings and a number of postsynaptic spikes. When a presynaptic action potential was paired with a single postsynaptic action potential at Δ*T* = 10 ms, the synapse underwent LTD for very low repetition frequencies and switched to LTP for the increasing frequency above 5 Hz (Figure 4A). A single postsynaptic spike, paired with the input activity at Δ*T* = 10 ms, induced LTD, while two, three, and four postsynaptic spikes led to LTP (Figure 4B). The results qualitatively reproduces the experimental observations on LTP recovery with increasing pairing frequency and postsynaptic spike number (Inglebert et al., 2020).

**Figure 5:**
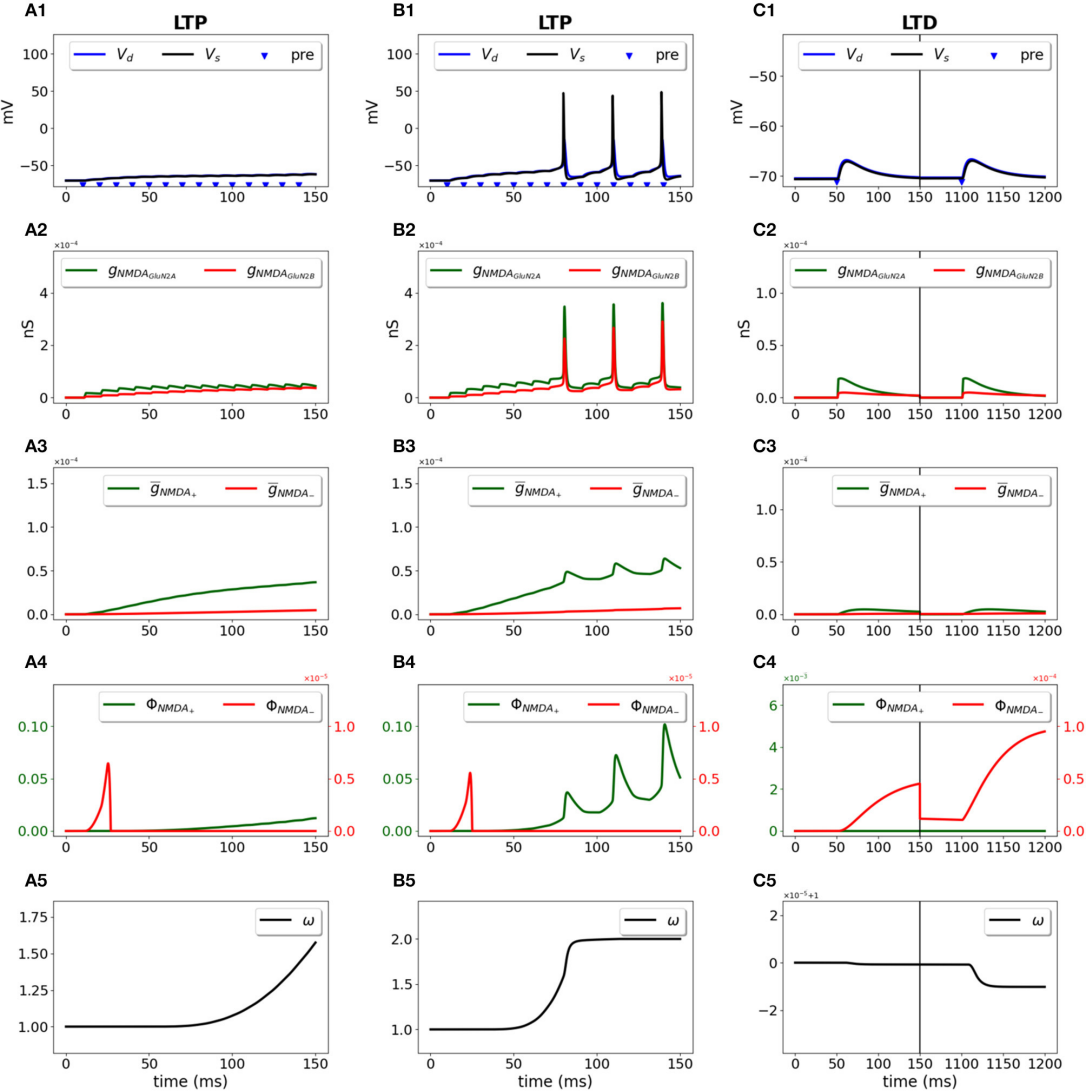
Synaptic weight change for the frequency-dependent subthreshold LTP **(A1–A5)**, suprathreshold LTP **(B1–B5)**, and LTD **(C1–C5)** protocols using a two-compartment model of CA1 pyramidal neuron. Presynaptic input was stimulated at 100 Hz for 1 s (LTP protocol) or at 1 Hz for 100 s (LTD protocol). **(A1–C1)** Membrane potential in soma *V*_*s*_ (black line) and dendrite *V*_*d*_ (blue line); presynaptic activity is indicated by blue triangles; **(A2–C2)** GluN2A-NMDAR and GluN2B-NMDAR conductances *g*_*NMD*_*A*__*GluN*2*A*__ (red line) and *g*_*NMD*_*A*__*GluN*2*B*__ (green line); **(A3–C3)** filtered NMDAR-dependent variables ${\bar{g}}_{NMDA_{-}}$ (red) and ${\bar{g}}_{NMDA_{+}}$ (green) for LTD and LTP components; **(A4–C4)** NMDAR-dependent functions ϕ_*NMD*_*A*__+__ (green line) and ϕ_*NMD*_*A*__−__ (red line) for the LTP and LTD components; **(A5–C5)** Synaptic weight *w*.

Next, we applied the frequency-dependent stimulation protocol using the same two-compartment model of CA1 pyramidal neuron with a single synapse (Figure 5). In subthreshold regime (Figures 5A1–A5), stimulation of presynaptic input at 100 Hz resulted in opening of Glu2NA-NMDAR and Glu2NB-NMDAR channels (Figure 5A2), activation of ϕ_*NMD*_*A*__+__ and inhibition of ϕ_*NMD*_*A*__−__ (Figures 5A3, A4, green and red lines respectively), and increase in weight *w* (Figure 5A5). In suprathreshold regime (Figures 5B1–B5), the same protocol led to the generation of somatic action potentials (Figure 5B1, black line), high activity of NMDAR channels, strong increase in ϕ_*NMD*_*A*__+__, and inhibition of ϕ_*NMD*_*A*__−__ (Figure 5B4) causing strong LTP (Figure 5B5). Low frequency stimulation at 1 Hz only slightly opened Glu2NA-NMDAR and Glu2NB-NMDAR channels (Figure 5C2) that was sufficient to activate ϕ_*NMD*_*A*__−__, but not ϕ_*NMD*_*A*__+__ (Figure 5C4, red line) and induce LTD (Figure 5C5). The results indicate that the model is suitable to account for the synaptic changes using frequency dependent LTD and LTP protocols in subthreshold and suprathreshold regimes.

The model validation analysis showed that the NMDAR-dependent synaptic plasticity model was capable of reproducing the experimental STDP weight change curves for different frequencies and postsynaptic patterns, high-frequency, and low-frequency stimulation protocols.

### 3.2. Synaptic plasticity depends on GluN2B-NMDAR properties in a synapse cluster on a CA1 pyramidal neuron

We employed the developed model of synaptic plasticity and a compartmental detailed model of a CA1 pyramidal neuron (Migliore et al., 2018) to analyze the dependence of synaptic modifications on the GluN2B-NMDAR functioning using STDP and frequency-dependent stimulation protocols. We modeled weight modifications at clustered synapses on the apical branches of CA1 pyramidal neuron and measured EPSP change in soma before and after the stimulation protocol. Experimental data and computational modeling studies suggest that synapses tend to form tightly-packed groups or clusters on the dendrites of neurons [for review see Kastellakis and Poirazi (2019) and Miry et al. (2021)]. Such nearly-synchronous activated inputs carry similar information onto the same dendrite and enable emerging of memory engrams. Thus, we formed a cluster of 50 effectively activated AMPAR and GluN2A-NMDAR/GluN2B-NMDAR containing synapses on the apical dendrites of CA1 pyramidal neuron.

**Figure 6:**
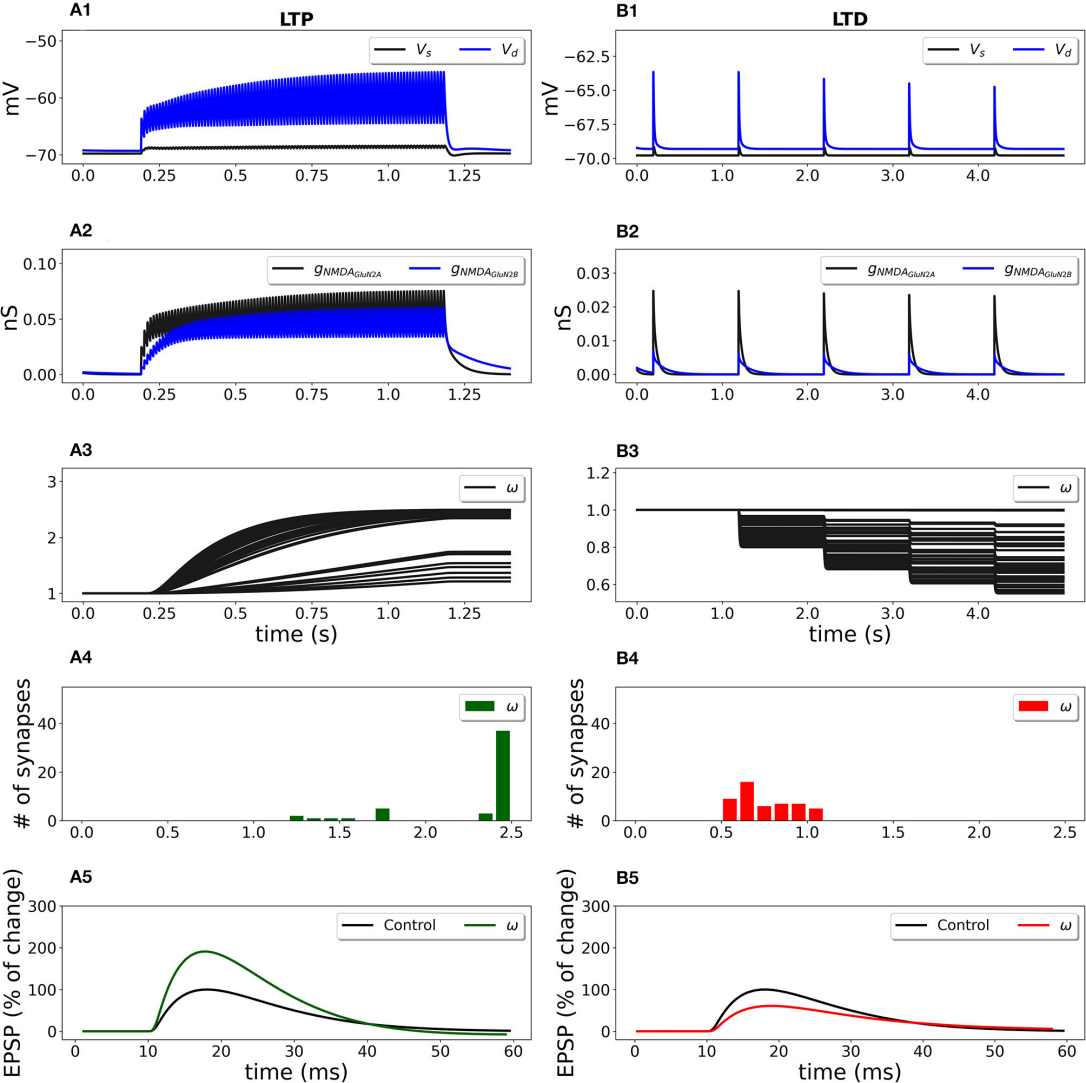
Synaptic weight change for the frequency-dependent LTP **(A1–A5)** and LTD **(B1–B5)** protocols using a multicompartmental model of CA1 pyramidal neuron. Fifty synapses were randomly distributed on the SR apical dendritic branches with the density of 0.8 synapses/μ*m* of dendrite 140 μ*m* from soma and stimulated at 100 Hz for 1 s (LTP protocol) and at 1 Hz for 100 s (LTD protocol). **(A1, B1)** Membrane potential in soma *V*_*s*_ (black line) and membrane potential *V*_*d*_ in dendrite (blue line) at a randomly selected synapse location; **(A2, B2)** GluN2A-NMDAR and GluN2B-NMDAR conductances *g*_*NMD*_*A*__*GluN*2*A*__ (black line) and *g*_*NMD*_*A*__*GluN*2*B*__ (blue line); **(A3, B3)** synaptic weights *w* of 50 synapses; **(A4, B4)** distribution of final synaptic weights *w*; **(A5)** normalized somatic EPSP before (black line) and after (green line) the synaptic plasticity induction protocol; LTP was induced with the EPSP change of 190%; **(B5)** normalized somatic EPSP before (black line) and after (red line) the synaptic plasticity induction protocol; LTD was triggered with the EPSP change of 61%.

During stimulation, each synapse on the dendritic branch developed its weight depending on the NMDAR-gated synaptic conductance function (Equation 4) that sensed local depolarization and presynaptic glutamate release. Figure 6 shows the evolvement of 50 synaptic weights in a cluster during LTP and LTD stimulation protocols.

High frequency presynaptic stimulation at 100 Hz for 1 s depolarized membrane potential (Figure 6A1; blue line—membrane potential *V*_*d*_ at a location of a randomly chosen synapse; black line—membrane potential in soma *V*_*s*_) and activated GluN2A-NMDAR and GluN2B-NMDAR channels *g*_*NMD*_*A*__*GluN*2*A*__ and *g*_*NMD*_*A*__*GluN*2*B*__ (Figure 6A2, black and blue lines, respectively) that led to the increase of synaptic weights (Figure 6A3). The histograms of the synaptic weights (Figure 6A4) shows that the weights distributed in the interval from 1 up to the predefined maximum value *w*_*max*_ = 2.5. Some synapses were only slightly potentiated due to the low local membrane potential and weakly activated NMDAR. The EPSP increased by 190% if compared to the EPSP before the conditioning stimulation (Figure 6A5, green line vs. black line). Low-frequency stimulation at 1 Hz for 100 s induced a small membrane depolarization at the synapse location (Figure 6B1, red line), weak NMDAR activation (Figure 6B2), and resulted in the weakening of synaptic strength of all synapses (Figures 6B3, B4). After the stimulation, the somatic EPSP decreased to 61% (Figure 6B5).

The synaptic plasticity model embedded into a detailed model of a CA1 pyramidal cell qualitatively reproduced the experimental results. Experimental data showed that 500 pulses at 1 Hz induced 57% LTD and 100 pulses at 100 Hz led to 191% LTP in hippocampal CA1 pyramidal neurons in rats (Pousinha et al., 2017).

**Figure 7:**
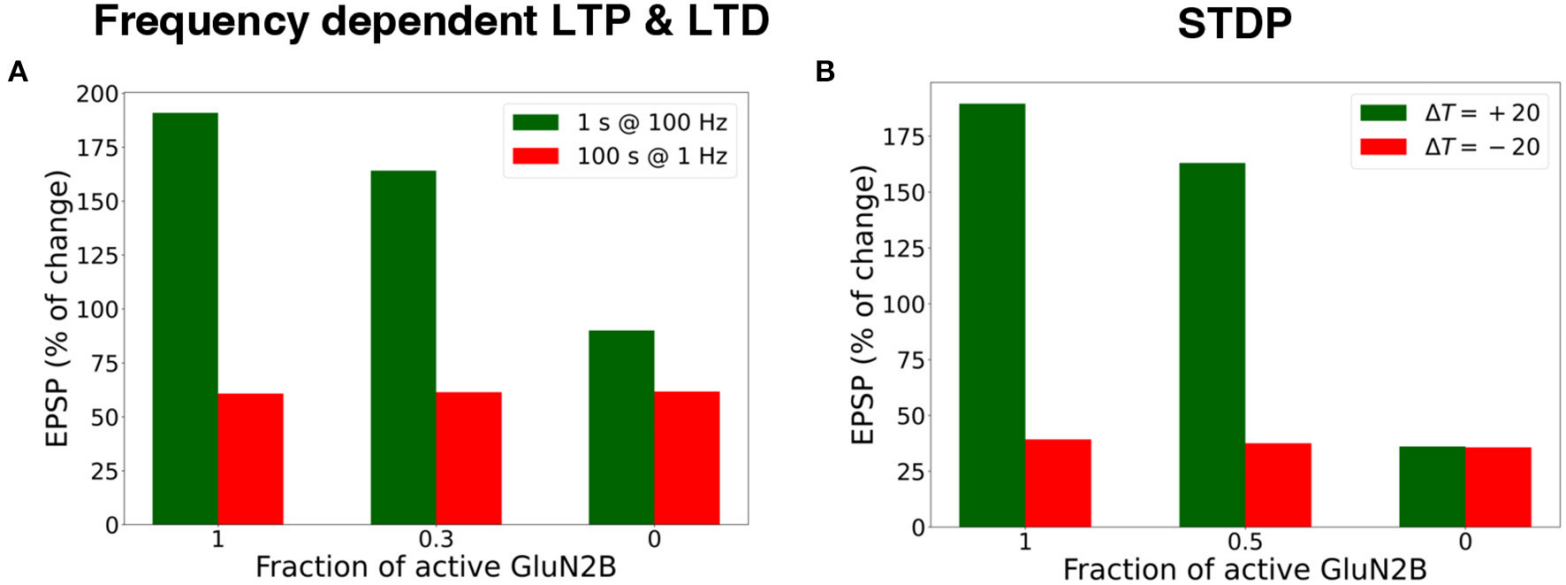
Blockade of GluN2B-NMDAR prevents LTP for high-frequency and STDP stimulation protocols. **(A)** Presynaptic input was stimulated at 100 Hz for 1 s (LTP protocol, green bars) and at 1 Hz for 100 s (LTD protocol, red bars). LTP was impaired, and LTD remained intact. **(B)** Presynaptic activity was paired with a doublet of postsynaptic action potentials 60 times at 5 Hz frequency with a temporal difference Δ*T* = +20 ms (LTP protocol, green bars) and Δ*T* = -20 ms (LTD protocol, red bars). LTP was abolished, and LTD was preserved.

We investigated the influence of the partial and full blockade of GluN2B-NMDAR on synaptic plasticity outcome using frequency-dependent stimulation and STDP protocols (Figure 7). First, presynaptic input was stimulated at 100 Hz for 1 s (LTP protocol), and the normalized EPSP to the pre-LTP baseline value was estimated. The blockade of the GluN2B-NMDAR synaptic conductance, leaving 0.3 fraction its active baseline value, resulted in the decrease of LTP from 190% to 164%, while the full blockade of the GluN2B-NMDAR led to LTD, the decrease to 90% of somatic EPSP. The impairment of GluN2B-NMDAR did not affect LTD leaving it to 63% (Figure 7A). The model of synaptic plasticity qualitatively reproduced experimental data of GluN2B-NMDAR inhibitor ifenprodil effect on LTP (Pousinha et al., 2017). It was shown that ifenprodil dose-dependently inhibited LTP, evoked by high frequency stimulation in CA3-CA1 synapses. The maximal inhibition efficacy was observed with the increasing ifenprodil concentration of 5 μ*m* that converted 190% LTP to 75% LTD, but it did not affect LTD. The results show that the synaptic plasticity model can capture the influence of GluN2B-NMDAR properties at a single synapse by decreasing the synaptic weight changes in response to the impaired GluN2B-NMDAR functioning, and quantitatively follows the experimental data (Pousinha et al., 2017).

Next, we applied the STDP stimulation protocol with the temporal difference Δ*T* = ± 20 ms between a presynaptic activity and a second postsynaptic spike (Figure 7B). GluN2B-NMDAR blockade led to the LTP switch into LTD for pre-post pairings and left LTD intact.

The results illustrate that synaptic plasticity is strongly affected by Glu2NB-NMDAR subunit properties. Hypofunction of GluN2B-NMDAR abolishes LTP induction and does not affect LTD for STDP protocol. For high frequency stimulation, LTP switches to LTD, and leaves LTD intact for low frequency stimulation. The results quantitatively align well with the experimental evidence on Glu2NB-NMDAR importance in shaping LTP at hippocampal synapse (Morishita et al., 2007; Andrade-Talavera et al., 2016; Pousinha et al., 2017). GluN2B-NMDAR may act as an additional modulatory mechanism of synaptic plasticity, and further experimental and computational studies are needed to understand the importance of the NMDAR subunit composition, and its effect on synaptic plasticity.

## 4. Discussion

We developed an NMDAR subunit-dependent voltage-based synaptic plasticity model of synaptic weight modifications at hippocampal CA3-CA1 synapses. We extended the computational model of STDP (Clopath et al., 2010; Meissner-Bernard et al., 2020) by simultaneously incorporating the GluN2A-NMDAR and GluN2B-NMDAR components to account for the specific functions of NMDAR subunits in synaptic learning. The model of synaptic plasticity was validated against the experimental data (Wittenberg and Wang, 2006; Pousinha et al., 2017; Inglebert et al., 2020) and reproduced STDP and frequency dependent LTP and LTD. Furthermore, the results show that this synaptic plasticity model is able to account for the impairment of LTP in GluN2B-NMDAR hypofunction conditions as in experimental studies (Pousinha et al., 2017), demonstrating that synaptic plasticity depends on GluN2B-NMDAR properties, and dysfunction of GluN2B-NMDAR leads to LTP impairment and its transformation to LTD.

The developed model can be interpreted as a phenomenological model, standing at the intersection with the class of biophysical models of synaptic plasticity. The model captures the influence of the GluN2B-NMDAR subunit on synaptic modifications. Usually, the calcium-based models of synaptic plasticity do not distinguish between the NMDAR subunits as mediators of calcium influx. Our approach enriches the model with the new features of specific NMDAR effect on synaptic plasticity.

Our model uses the functions of the NMDAR subunit dynamics that in an abstract form accounts for the CaMKII and phosphatase activation, does not require to model dendritic spines and estimate intracellular calcium concentration, a main trigger of synaptic plasticity. The formalism proposed captures synapse-specific mechanisms that define synaptic plasticity—the local non-linear activation of NMDAR, its subunit composition and functioning.

We chose the voltage-based approach as the voltage traces are conventionally recorded in the experimental setups, well-described by the mathematical formalism, enabling the model to be usable for future network level simulations. The level of modeling may also be more detailed and rely on intracellular calcium dynamics, as in e.g., Shouval et al. (2002) and Graupner and Brunel (2012), while focusing on the NMDAR subunit effects on LTP and LTD induction.

We explored the impact of the NMDAR properties on the somatic EPSP changes using a biologically realistic multicompartmental CA1 pyramidal neuron and taking into account the influence of the spatial distribution of the synapses. The results indicate that GluN2B-NMDAR regulates the amount of synaptic strength on the dendritic tree and the resulting EPSP changes in soma. The hypofunction of GluN2B-NMDAR leads to the impairment of LTP and gradual switch to LTD. The study extends the experimental observations of Pousinha et al. (2017) and predicts the pattern of the GluN2B-NMDAR functioning-mediated synaptic plasticity measured as changes in somatic EPSP for specific synapse cluster for STDP induction protocol. In detailed biophysical modeling studies, long-term synaptic plasticity depends on intracellular calcium influx, but the sources of calcium is usually not taken into consideration. Here, we discuss the importance of considering the mediating role of Glu2NB-NMDAR to study LTP in STDP and frequency dependent synaptic plasticity. As NMDARs can undergo activity-dependent long-term plasticity (Hunt and Castillo, 2012), this work shows the importance to consider the state of NMDARs, not only in the modeling studies of learning and memory, but also in physiological experiments. The model offers a possibility to include GluN2B-NMDAR effective contribution to the synaptic weight modifications. The incorporation of the separated influence of the GluN2A-NMDAR and GluN2B-NMDAR functioning makes the model a candidate to explore learning in the diseased brain, as the Glu2NB-NMDAR normal functioning is crucial for healthy CA3-CA1 synapses, and its dysfunction is observed in cognitive deficits in neurological diseases (Kocsis, 2012; Pousinha et al., 2017, 2019; Adell, 2020).

The model of synaptic plasticity is principally based on the critical role of postsynaptic NMDAR in LTP and LTD induction in adult CA3-CA1 synapses. GluN2A-NMDAR and GluN2B-NMDAR subunits mediate some forms of LTP and LTD at CA3-CA1 synapses (Paoletti et al., 2013). Experimental evidence suggests that GluN2B-NMDAR subunits are critical for LTP, but not necessary for LTD (Weitlauf et al., 2005; Bartlett et al., 2007). GluN2A-NMDAR blockade prevented LTD induction in the CA1 region of hippocampal slices (Bartlett et al., 2007; Li et al., 2007). However, other studies found that loss of GluN2B-NMDAR prevented LTD (Brigman et al., 2010), and GluN2A-NMDAR is not necessary for LTD (Gerkin et al., 2007; Li et al., 2007; Ge et al., 2010). Studies on GluN2 subunit composition for LTD have been inconsistent and conflicting, likely due to the problematic GluN2 subunit-selective pharmacology (Neyton and Paoletti, 2006; Shipton and Paulsen, 2014; Wong and Gray, 2018; Franchini et al., 2020). Sometimes seemingly the contradicting experimental data of synaptic plasticity outcomes depend on the developmental stage of the animal, extracellular or intracellular solution compositions, and other variables linked to the different experimental settings. In general, it is hypothesized that the GluN2A-to-GluN2B ratio defines the magnitude and sign of frequency-induced synaptic plasticity and shifts the LTP and LTD threshold. Higher GluN2A-to-GluN2B ratio requires stronger stimulation to induce LTP, confirming the critical role of GluN2B in LTP (Paoletti et al., 2013).

Recent experimental evidence shows that synaptic plasticity has different induction mechanisms depending on the NMDAR position (pre- or post-synaptic) and subunit composition, developmental stage of animal, or experimental settings including the type of protocol used. For example, presynaptic NMDARs at the CA3-CA1 synapse can mediate a pre-synaptic form of STDP LTD (t-LTD) in young mice (P13-021) (Andrade-Talavera et al., 2016). This t-LTD is lost in adult synapses when applying the same post-pre protocol and even converts LTP in adult animals not requiring NMDARs anymore (Pérez-Rodríguez et al., 2019; Falcón-Moya et al., 2020). Such diversity in mechanisms of synapse plasticity, even within one type of synapse, shows the limitation of the synaptic plasticity model proposed and indicates the need to extend the study by including other mechanisms such as the involvement of presynaptic NMDAR, group I metabotropic glutamate receptor (mGluR), astrocytic signaling.

The limitation of this study is the phenomenological nature of the synaptic plasticity model that relies on the NMDAR subunit-dependent synaptic plasticity functions, but does not include detailed molecular pathways of the possible LTP and LTD induction mechanisms such as protein kinase A (PKA), CaMKII, protein phosphatase 2A and 2B (PP2A, PP2B) activation and competition. The potential direction of synaptic plasticity studies is the extension of the detailed biophysical models to account for the influence of the postsynaptic NMDAR subunit effects on the biochemical pathways of CaMKII, PKA, PP2A/2B activation in LTP and LTD induction. On the other hand, the model allows reducing the complexity of the description of the molecular network underlying LTP and LTD. We were also confronted with sometimes seemingly contradicting experimental data of synaptic outcomes that probably depend on the developmental stage of the animal, extracellular or intracellular solution compositions, and other variables linked to the different experimental settings. For example, pre-post pairings of synaptic activity lead to LTD for 5 Hz stimulation (Wittenberg and Wang, 2006), but to LTP in Inglebert et al. (2020), and it might be explained by the extracellular calcium concentration (Inglebert et al., 2020).

The model could in future studies be extended to include more complexity and account not only for postsynaptic Glu2NA NMDAR and Glu2NB-NMDAR, but also for the influence of mGluR activation, endocannabinoind, astrocytic signaling, presynaptic Glu2NC/D-NMDAR mediating effect on synaptic plasticity (Andrade-Talavera et al., 2016), adenosine (Pérez-Rodríguez et al., 2019), or other possible postsynaptic and non-postsynaptic mediators.

Current trend in neuroscience is shifting the focus toward studying the relationships between different levels and scales of brain organization to better understand the nervous systems and, ultimately, human behavior. Strong emphasis in the field is to connect these different levels and scales by multiscale techniques, to allow better exploration of the information flow between the cellular-, molecular-, and network/circuit-level phenomena, and the cognitive processes and behavior. Therefore, the presented model of synaptic plasticity that enables linking synapse-specific NMDAR function to the cell and network behavior is an important step toward understanding learning in hippocampal networks.

## Data availability statement

The original contributions presented in the study are included in the article/Supplementary material, further inquiries can be directed to the corresponding author.

## Author contributions

AS, MM, JD, and HM planned the research. AS and JD performed the numerical simulations and wrote the manuscript. All authors have read and approved the final manuscript.

## References

[B1] Adell A. (2020). Brain NMDA receptors in schizophrenia and depression. Biomolecules 10:947. 10.3390/biom10060947 32585886 PMC7355879

[B2] Andrade-Talavera Y. Duque-Feria P. Paulsen O. Rodríguez-Moreno A. (2016). Presynaptic spike timing-dependent long-term depression in the mouse hippocampus. Cereb. Cortex 26, 3637–3654. 10.1093/cercor/bhw172 27282393 PMC4961031

[B3] Badoual M. Zou Q. Davison A. Lilith M. Bal T. Frégnac Y.. (2006). Biophysical and phenomenological models of multiple spike interactions in spike-timing dependent plasticity. Int. J. Neural Syst. 16, 79–97. 10.1142/S0129065706000524 16688849

[B4] Bartlett T. E. Bannister N. J. Collett V. J. Dargan S. L. Massey P. V. Bortolotto Z. A.. (2007). Differential roles of NR2A and NR2B-containing NMDA receptors in LTP and LTD in the CA1 region of two-week old rat hippocampus. Neuropharmacology 52, 60–70. 10.1016/j.neuropharm.2006.07.013 16904707

[B5] Berberich S. Punnakkal P. Jensen V. Pawlak V. Seeburg P. H. Hvalby Ø.. (2005). Lack of NMDA receptor subtype selectivity for hippocampal long-term potentiation. J. Neurosci. 25, 6907–6910. 10.1523/JNEUROSCI.1905-05.2005 16033900 PMC6725356

[B6] Bezaire M. J. Raikov I. Burk K. Vyas D. Soltesz I. (2016). Interneuronal mechanisms of hippocampal theta oscillations in a full-scale model of the rodent CA1 circuit. Elife 5:e18566. 10.7554/eLife.18566 28009257 PMC5313080

[B7] Bhalla U. S. Iyengar R. (1999). Emergent properties of networks of biological signaling pathways. Science 283, 381–387. 10.1126/science.283.5400.381 9888852

[B8] Bi G.-Q. Poo M.-M. (1998). Synaptic modifications in cultured hippocampal neurons: dependence on spike timing, synaptic strength, and postsynaptic cell type. J. Neurosci. 18, 10464–10472. 10.1523/JNEUROSCI.18-24-10464.1998 9852584 PMC6793365

[B9] Bliss T. V. Collingridge G. L. (2013). Expression of NMDA receptor-dependent LTP in the hippocampus: bridging the divide. Mol. Brain 6, 5. 10.1186/1756-6606-6-5 23339575 PMC3562207

[B10] Bliss T. V. P. Collingridge G. L. (1993). A synaptic model of memory: long-term potentiation in the hippocampus. Nature 361, 31–39. 10.1038/361031a0 8421494

[B11] Brigman J. L. Wright T. Talani G. Prasad-Mulcare S. Jinde S. Seabold G. K.. (2010). Loss of GluN2B-containing NMDA receptors in CA1 hippocampus and cortex impairs long-term depression, reduces dendritic spine density, and disrupts learning. J. Neurosci. 30, 4590–4600. 10.1523/JNEUROSCI.0640-10.2010 20357110 PMC2869199

[B12] Buzsáki G. (2002). Theta oscillations in the hippocampus. Neuron 33, 325–340. 10.1016/S0896-6273(02)00586-X 11832222

[B13] Chindemi G. Abdellah M. Amsalem O. Benavides-Piccione R. Delattre V. Doron M.. (2022). A calcium-based plasticity model for predicting long-term potentiation and depression in the neocortex. Nat. Commun. 13, 3038. 10.1038/s41467-022-30214-w 35650191 PMC9160074

[B14] Clayton D. A. Mesches M. H. Alvarez E. Bickford P. C. Browning M. D. (2002). A hippocampal NR2B deficit can mimic age-related changes in long-term potentiation and spatial learning in the Fischer 344 rat. J. Neurosci. 22, 3628–3637. 10.1523/JNEUROSCI.22-09-03628.2002 11978838 PMC6758397

[B15] Clopath C. Büsing L. Vasilaki E. Gerstner W. (2010). Connectivity reflects coding: a model of voltage-based STDP with homeostasis. Nat. Neurosci. 13, 344–352. 10.1038/nn.2479 20098420

[B16] Collingridge G. L. Bliss T. V. P. (1995). Memories of NMDA receptors and LTP. Trends Neurosci. 18, 54–56. 10.1093/acprof:oso/9780192625021.001.0001 7537406

[B17] Collingridge G. L. Kehl S. J. McLennan H. (1983). Excitatory amino acids in synaptic transmission in the Schaffer collateral-commissural pathway of the rat hippocampus. J. Physiol. 334, 33–46. 10.1113/jphysiol.1983.sp014478 6306230 PMC1197298

[B18] Cui Y. Jin J. Zhang X. Xu H. Yang L. Du D.. (2011). Forebrain NR2B overexpression facilitating the prefrontal cortex long-term potentiation and enhancing working memory function in mice. PLoS ONE 6:e20312. 10.1371/journal.pone.0020312 21655294 PMC3105019

[B19] Cull-Candy S. Brickley S. Farrant M. (2001). NMDA receptor subunits: diversity, development and disease. Curr. Opin. Neurobiol. 11, 327–335. 10.1016/S0959-4388(00)00215-4 11399431

[B20] Debanne D. Gähwiler B. H. Thompson S. M. (1998). Long-term synaptic plasticity between pairs of individual CA3 pyramidal cells in rat hippocampal slice cultures. J. Physiol. 507(Pt 1), 237–247. 10.1111/j.1469-7793.1998.237bu.x 9490845 PMC2230782

[B21] Destexhe A. Mainen Z. F. Sejnowski T. J. (1994). Synthesis of models for excitable membranes, synaptic transmission and neuromodulation using a common kinetic formalism. J. Comput. Neurosci. 1, 195–230. 10.1007/BF00961734 8792231

[B22] Dudek S. M. Bear M. F. (1992). Homosynaptic long-term depression in area CA1 of hippocampus and effects of N-methyl-D-aspartate receptor blockade. Proc. Natl. Acad. Sci. U.S.A. 89, 4363–4367. 10.1073/pnas.89.10.4363 1350090 PMC49082

[B23] Ebner C. Clopath C. Jedlicka P. Cuntz H. (2019). Unifying long-term plasticity rules for excitatory synapses by modeling dendrites of cortical pyramidal neurons. Cell Rep. 29, 4295.e6–4307.e6. 10.1016/j.celrep.2019.11.068 31875541 PMC6941234

[B24] Falcón-Moya R. Pérez-Rodríguez M. Prius-Mengual J. Andrade-Talavera Y. Arroyo-García L. E. Pérez-Artés R.. (2020). Astrocyte-mediated switch in spike timing-dependent plasticity during hippocampal development. Nat. Commun. 11, 4388. 10.1038/s41467-020-18024-4 32873805 PMC7463247

[B25] Feldman D. E. (2012). The spike-timing dependence of plasticity. Neuron 75, 556–571. 10.1016/j.neuron.2012.08.001 22920249 PMC3431193

[B26] Ferguson K. Campbell S. (2009). A two compartment model of a CA1 pyramidal neuron. Canad. Appl. Math. Quart. 17, 293–307.

[B27] Foster K. A. McLaughlin N. Edbauer D. Phillips M. Bolton A. Constantine-Paton M.. (2010). Distinct roles of NR2A and NR2B cytoplasmic tails in long-term potentiation. J. Neurosci. 30, 2676–2685. 10.1523/JNEUROSCI.4022-09.2010 20164351 PMC2840640

[B28] France G. Fernández-Fernández D. Burnell E. S. Irvine M. W. Monaghan D. T. Jane D. E.. (2017). Multiple roles of GluN2B-containing NMDA receptors in synaptic plasticity in juvenile hippocampus. Neuropharmacology 112(Pt A), 76–83. 10.1016/j.neuropharm.2016.08.010 27523302 PMC5084684

[B29] Franchini L. Carrano N. Di Luca M. Gardoni F. (2020). Synaptic GluN2A-containing NMDA receptors: from physiology to pathological synaptic plasticity. Int. J. Mol. Sci. 21, 1538. 10.3390/ijms21041538 32102377 PMC7073220

[B30] Gardoni F. Mauceri D. Malinverno M. Polli F. Costa C. Tozzi A.. (2009). Decreased NR2B subunit synaptic levels cause impaired long-term potentiation but not long-term depression. J. Neurosci. 29, 669–677. 10.1523/JNEUROSCI.3921-08.2009 19158293 PMC6665154

[B31] Gasparini S. Migliore M. Magee J. C. (2004). On the initiation and propagation of dendritic spikes in CA1 pyramidal neurons. J. Neurosci. 24, 11046–11056. 10.1523/JNEUROSCI.2520-04.2004 15590921 PMC6730267

[B32] Ge Y. Dong Z. Bagot R. C. Howland J. G. Phillips A. G. Wong T. P.. (2010). Hippocampal long-term depression is required for the consolidation of spatial memory. Proc. Natl. Acad. Sci. U.S.A. 107, 16697–16702. 10.1073/pnas.1008200107 20823230 PMC2944752

[B33] Gerkin R. C. Lau P.-M. Nauen D. W. Wang Y. T. Bi G.-Q. (2007). Modular competition driven by NMDA receptor subtypes in spike-timing-dependent plasticity. J. Neurophysiol. 97, 2851–2862. 10.1152/jn.00860.2006 17267756

[B34] Gerstner W. Kempter R. van Hemmen J. L. Wagner H. (1996). A neuronal learning rule for sub-millisecond temporal coding. Nature 383, 76–78. 10.1038/383076a0 8779718

[B35] Goh J. J. Manahan-Vaughan D. (2013). Synaptic depression in the CA1 region of freely behaving mice is highly dependent on afferent stimulation parameters. Front. Integr. Neurosci. 7:1. 10.3389/fnint.2013.00001 23355815 PMC3555076

[B36] Graupner M. Brunel N. (2007). STDP in a bistable synapse model based on CaMKII and associated signaling pathways. PLOS Computat. Biol. 3:e221. 10.1371/journal.pcbi.0030221 18052535 PMC2098851

[B37] Graupner M. Brunel N. (2012). Calcium-based plasticity model explains sensitivity of synaptic changes to spike pattern, rate, and dendritic location. Proc. Nat. Acad. Sci. U.S.A. 109, 3991–3996. 10.1073/pnas.1109359109 22357758 PMC3309784

[B38] Hines M. L. Carnevale N. T. (1997). The NEURON simulation environment. Neural Comput. 9, 1179–1209. 10.1162/neco.1997.9.6.1179 9248061

[B39] Hunt D. L. Castillo P. E. (2012). Synaptic plasticity of NMDA receptors: mechanisms and functional implications. Curr. Opin. Neurobiol. 22, 496–508. 10.1016/j.conb.2012.01.007 22325859 PMC3482462

[B40] Inglebert Y. Aljadeff J. Brunel N. Debanne D. (2020). Synaptic plasticity rules with physiological calcium levels. Proc. Natl. Acad. Sci. U.S.A. 117, 33639–33648. 10.1073/pnas.2013663117 33328274 PMC7777146

[B41] Jędrzejewska-Szmek J. Damodaran S. Dorman D. B. Blackwell K. T. (2017). Calcium dynamics predict direction of synaptic plasticity in striatal spiny projection neurons. Eur. J. Neurosci. 45, 1044–1056. 10.1111/ejn.13287 27233469 PMC5124545

[B42] Kastellakis G. Poirazi P. (2019). Synaptic Clustering and Memory Formation. Front. Mol. Neurosci. 12:300. 10.3389/fnmol.2019.00300 31866824 PMC6908852

[B43] Kempter R. Gerstner W. van Hemmen J. L. (1999). Hebbian learning and spiking neurons. Phys. Rev. E 59, 4498–4514. 10.1103/PhysRevE.59.4498

[B44] Kistler W. van Hemmen L. (2000). Modeling synaptic plasticity in conjunction with the timing of pre- and postsynaptic action potentials. Neural Comput. 12, 385–405. 10.1162/089976600300015844 10636948

[B45] Kocsis B. (2012). Differential role of NR2A and NR2B subunits in N-methyl-D-aspartate receptor antagonist-induced aberrant cortical gamma oscillations. Biol. Psychiatry 71, 987–995. 10.1016/j.biopsych.2011.10.002 22055014 PMC3276718

[B46] Lau B. Cole S. R. Gange S. J. (2009). Competing risk regression models for epidemiologic data. Am. J. Epidemiol. 170, 244–256. 10.1093/aje/kwp107 19494242 PMC2732996

[B47] Li R. Huang F.-S. Abbas A.-K. Wigström H. (2007). Role of NMDA receptor subtypes in different forms of NMDA-dependent synaptic plasticity. BMC Neurosci. 8, 55. 10.1186/1471-2202-8-55 17655746 PMC1959237

[B48] Liu J. Chang L. Song Y. Li H. Wu Y. (2019). The role of NMDA receptors in Alzheimer's disease. . Front. Neurosci. 13:43. 10.3389/fnins.2019.00043 30800052 PMC6375899

[B49] Liu L. Wong T. P. Pozza M. F. Lingenhoehl K. Wang Y. Sheng M.. (2004). Role of NMDA receptor subtypes in governing the direction of hippocampal synaptic plasticity. Science 304, 1021–1024. 10.1126/science.1096615 15143284

[B50] Lüscher C. Malenka R. C. (2012). NMDA receptor-dependent long-term potentiation and long-term depression (LTP/LTD). Cold Spring Harb. Perspect. Biol. 4, a005710. 10.1101/cshperspect.a005710 22510460 PMC3367554

[B51] MacDermott A. B. Mayer M. L. Westbrook G. L. Smith S. J. Barker J. L. (1986). NMDA-receptor activation increases cytoplasmic calcium concentration in cultured spinal cord neurones. Nature 321, 519–522. 10.1038/321519a0 3012362

[B52] MacDonald S. W. S. Nyberg L. Bäckman L. (2006). Intra-individual variability in behavior: links to brain structure, neurotransmission and neuronal activity. Trends Neurosci. 29, 474–480. 10.1016/j.tins.2006.06.011 16820224

[B53] Mäki-Marttunen T. Iannella N. Edwards A. G. Einevoll G. T. Blackwell K. T. (2020). A unified computational model for cortical post-synaptic plasticity. eLife 9, e55714. 10.7554/eLife.55714.sa2 32729828 PMC7426095

[B54] Malenka R. C. Bear M. F. (2004). LTP and LTD: an embarrassment of riches. Neuron 44, 5–21. 10.1016/j.neuron.2004.09.012 15450156

[B55] Malenka R. C. Nicoll R. A. (1999). Long-term potentiation–a decade of progress? Science 285, 1870–1874. 10.1126/science.285.5435.1870 10489359

[B56] Markram H. Lübke J. Frotscher M. Roth A. Sakmann B. (1997). Physiology and anatomy of synaptic connections between thick tufted pyramidal neurones in the developing rat neocortex. J. Physiol. 500(Pt 2), 409–440. 10.1113/jphysiol.1997.sp022031 9147328 PMC1159394

[B57] Meissner-Bernard C. Tsai M. C. Logiaco L. Gerstner W. (2020). Dendritic voltage recordings explain paradoxical synaptic plasticity: a modeling study. Front. Synaptic Neurosci. 12:585539. 10.3389/fnsyn.2020.585539 33224033 PMC7670913

[B58] Migliore M. De Simone G. Migliore R. (2015). Effect of the initial synaptic state on the probability to induce long-term potentiation and depression. Biophys. J. 108, 1038–1046. 10.1016/j.bpj.2014.12.048 25762316 PMC4375721

[B59] Migliore R. Lupascu C. A. Bologna L. L. Romani A. Courcol J.-D. Antonel S.. (2018). The physiological variability of channel density in hippocampal CA1 pyramidal cells and interneurons explored using a unified data-driven modeling workflow. PLOS Comput. Biol. 14:e1006423. 10.1371/journal.pcbi.1006423 30222740 PMC6160220

[B60] Miry O. Li J. Chen L. (2021). The quest for the hippocampal memory engram: from theories to experimental evidence. Front. Behav. Neurosci. 14:632019. 10.3389/fnbeh.2020.632019 33519396 PMC7843437

[B61] Morishita W. Lu W. Smith G. B. Nicoll R. A. Bear M. F. Malenka R. C. (2007). Activation of NR2B-containing NMDA receptors is not required for NMDA receptor-dependent long-term depression. Neuropharmacology 52, 71–76. 10.1016/j.neuropharm.2006.07.005 16899258

[B62] Mulkey R. M. Malenka R. C. (1992). Mechanisms underlying induction of homosynaptic long-term depression in area CA1 of the hippocampus. Neuron 9, 967–975. 10.1016/0896-6273(92)90248-c 1419003

[B63] Neyton J. Paoletti P. (2006). Relating NMDA receptor function to receptor subunit composition: Limitations of the pharmacological approach. J. Neurosci. 26, 1331–1333. 10.1523/JNEUROSCI.5242-05.2006 16452656 PMC6675501

[B64] Paoletti P. (2011). Molecular basis of NMDA receptor functional diversity. Eur. J. Neurosci. 33, 1351–1365. 10.1111/j.1460-9568.2011.07628.x 21395862

[B65] Paoletti P. Bellone C. Zhou Q. (2013). NMDA receptor subunit diversity: impact on receptor properties, synaptic plasticity and disease. Nat. Rev. Neurosci. 14, 383–400. 10.1038/nrn3504 23686171

[B66] Parameshwaran K. Dhanasekaran M. Suppiramaniam V. (2008). Amyloid beta peptides and glutamatergic synaptic dysregulation. Exp. Neurol. 210, 7–13. 10.1016/j.expneurol.2007.10.008 18053990

[B67] Park P. Georgiou J. Sanderson T. M. Ko K.-H. Kang H. Kim J.-I.. (2021). PKA drives an increase in AMPA receptor unitary conductance during LTP in the hippocampus. Nat. Commun. 12, 413. 10.1038/s41467-020-20523-3 33462202 PMC7814032

[B68] Pérez-Rodríguez M. Arroyo-García L. E. Prius-Mengual J. Andrade-Talavera Y. Armengol J. A. Pérez-Villegas E. M.. (2019). Adenosine receptor-mediated developmental loss of spike timing-dependent depression in the hippocampus. Cereb. Cortex 29, 3266–3281. 10.1093/cercor/bhy194 30169759 PMC6644873

[B69] Pfister J.-P. Toyoizumi T. Barber D. Gerstner W. (2006). Optimal spike-timing-dependent plasticity for precise action potential firing in supervised learning. Neural Comput. 18, 1318–1348. 10.1162/neco.2006.18.6.1318 16764506

[B70] Pi H. J. Lisman J. E. (2008). Coupled phosphatase and kinase switches produce the tristability required for long-term potentiation and long-term depression. J. Neurosci. 28, 13132–13138. 10.1523/JNEUROSCI.2348-08.2008 19052204 PMC2620235

[B71] Pinsky P. F. Rinzel J. (1994). Intrinsic and network rhythmogenesis in a reduced Traub model for CA3 neurons. J. Comput. Neurosci. 1, 39–60. 10.1007/BF00962717 8792224

[B72] Poirazi P. Papoutsi A. (2020). Illuminating dendritic function with computational models. Nat. Rev. Neurosci. 21, 303–321. 10.1038/s41583-020-0301-7 32393820

[B73] Pousinha P. A. Mouska X. Bianchi D. Temido-Ferreira M. Rajão-Saraiva J. Gomes R.. (2019). The amyloid precursor protein C-terminal domain alters CA1 neuron firing, modifying hippocampus oscillations and impairing spatial memory encoding. Cell Rep. 29, 317.e5–331.e5. 10.1016/j.celrep.2019.08.103 31597094

[B74] Pousinha P. A. Mouska X. Raymond E. F. Gwizdek C. Dhib G. Poupon G.. (2017). Physiological and pathophysiological control of synaptic GluN2B-NMDA receptors by the C-terminal domain of amyloid precursor protein. eLife 6, e25659. 10.7554/eLife.25659.015 28682239 PMC5544428

[B75] Sacramento J. Costa R. P. Bengio Y. Senn W. (2018). Dendritic cortical microcircuits approximate the backpropagation algorithm, in Proceedings of the 32nd International Conference on Neural Information Processing Systems, NIPS'18 (Red Hook, NY: Curran Associates Inc.), 8735–8746.

[B76] Saudargiene A. Cobb S. Graham B. P. (2015). A computational study on plasticity during theta cycles at Schaffer collateral synapses on CA1 pyramidal cells in the hippocampus. Hippocampus 25, 208–218. 10.1002/hipo.22365 25220633

[B77] Senn W. Markram H. Tsodyks M. (2001). An algorithm for modifying neurotransmitter release probability based on pre- and post-synaptic spike timing. Neural Comput. 13, 35–67. 10.1162/089976601300014628 11177427

[B78] Shipton O. A. Paulsen O. (2014). GluN2A and GluN2B subunit-containing NMDA receptors in hippocampal plasticity. Philos. Trans. R. Soc. Lond. B Biol. Sci. 369, 20130163. 10.1098/rstb.2013.0163 24298164 PMC3843894

[B79] Shouval H. Z. Bear M. F. Cooper L. N. (2002). A unified model of NMDA receptor-dependent bidirectional synaptic plasticity. Proc. Natl. Acad. Sci. U.S.A. 99, 10831–10836. 10.1073/pnas.152343099 12136127 PMC125058

[B80] Song S. Abbott L. (2000). Temporally asymmetric Hebbian learning and neuronal response variability. Neurocomputing 32–33, 523–528. 10.1016/S0925-2312(00)00208-327534393

[B81] Song S. Miller K. D. Abbott L. F. (2000). Competitive Hebbian learning through spike-timing-dependent synaptic plasticity. Nat. Neurosci. 3, 919–926. 10.1038/78829 10966623

[B82] Standage D. Trappenberg T. Blohm G. (2014). Calcium-dependent calcium decay explains STDP in a dynamic model of hippocampal synapses. PLoS ONE 9:e86248. 10.1371/journal.pone.0086248 24465987 PMC3899242

[B83] Toyoizumi T. Pfister J.-P. Aihara K. Gerstner W. (2005). Generalized Bienenstock–Cooper–Munro rule for spiking neurons that maximizes information transmission. Proc. Nat. Acad. Sci. U.S.A. 102, 5239–5244. 10.1073/pnas.0500495102 15795376 PMC555686

[B84] Urbanczik R. Senn W. (2014). Learning by the dendritic prediction of somatic spiking. Neuron 81, 521–528. 10.1016/j.neuron.2013.11.030 24507189

[B85] Volianskis A. France G. Jensen M. S. Bortolotto Z. A. Jane D. E. Collingridge G. L. (2015). Long-term potentiation and the role of N-methyl-d-aspartate receptors. Brain Res. 1621, 5–16. 10.1016/j.brainres.2015.01.016 25619552 PMC4563944

[B86] Weitlauf C. Honse Y. Auberson Y. P. Mishina M. Lovinger D. M. Winder D. G. (2005). Activation of NR2A-containing NMDA receptors is not obligatory for NMDA receptor-dependent long-term potentiation. J. Neurosci. 25, 8386–8390. 10.1523/JNEUROSCI.2388-05.2005 16162920 PMC6725680

[B87] Wittenberg G. M. Wang S. S.-H. (2006). Malleability of spike-timing-dependent plasticity at the CA3-CA1 synapse. J. Neurosci. 26, 6610–6617. 10.1523/JNEUROSCI.5388-05.2006 16775149 PMC6674029

[B88] Wong J. M. Gray J. A. (2018). Long-term depression is independent of GluN2 subunit composition. J. Neurosci. 38, 4462–4470. 10.1523/JNEUROSCI.0394-18.2018 29593052 PMC5943974

[B89] Yasuda R. Hayashi Y. Hell J. W. (2022). CaMKII: a central molecular organizer of synaptic plasticity, learning and memory. Nat. Rev. Neurosci. 23, 666–682. 10.1038/s41583-022-00624-2 36056211

[B90] Zamzow D. R. Elias V. Shumaker M. Larson C. Magnusson K. R. (2013). An increase in the association of GluN2B containing NMDA receptors with membrane scaffolding proteins was related to memory declines during aging. J. Neurosci. 33, 12300–12305. 10.1523/JNEUROSCI.0312-13.2013 23884936 PMC3721840

[B91] Zhao M.-G. Toyoda H. Lee Y.-S. Wu L.-J. Ko S. W. Zhang X.-H.. (2005). Roles of NMDA NR2B subtype receptor in prefrontal long-term potentiation and contextual fear memory. Neuron 47, 859–872. 10.1016/j.neuron.2005.08.014 16157280

[B92] Zhou Y. Takahashi E. Li W. Halt A. Wiltgen B. Ehninger D.. (2007). Interactions between the NR2B receptor and CaMKII modulate synaptic plasticity and spatial learning. J. Neurosci. 27, 13843–13853. 10.1523/JNEUROSCI.4486-07.2007 18077696 PMC6673634

