# Supplementary material for: GluN2B-NMDAR subunit contribution on synaptic plasticity: A phenomenological model for CA3-CA1 synapses
Source: Front Synaptic Neurosci. 2023 Mar 15;15:1113957. doi: 10.3389/fnsyn.2023.1113957 (PMC10050887; doi:10.3389/fnsyn.2023.1113957)
Supplement: Supplementary file 1 [file Data_Sheet_1.pdf]

Supplementary Material

GluN2B-NMDAR subunit contribution on synaptic plasticity: A phenomenological model for CA3-CA1 synapses

Justinas J. Dainauskas [1,2], Hélène Marie [3], Michele Migliore [4], and Ausra Saudargiene [1,*]

[1]Neuroscience Institute, Lithuanian University of Health Sciences, Kaunas, Lithuania
[2]Department of Informatics, Vytautas Magnus University, Kaunas, Lithuania
[3]University Côte d'Azur, Centre National de la Recherche Scientifique (CNRS) UMR 7275, Institut de Pharmacologie Moléculaire et Cellulaire (IPMC), Valbonne, France
[4]Institute of Biophysics, National Research Council, Palermo, Italy

Corresponding author: ausra.saudargiene@lsmuni.lt

1 SUPPLEMENTARY DATA

NMDAR-dependent model of synaptic plasticity was validated using a two-compartment model (Pinsky and Rinzel, 1994; Ferguson and Campbell, 2009) and further analysed using a multicompartmental model of CA1 pyramidal neuron (Migliore et al., 2018). Spike-timing-dependent synaptic plasticity (STDP) (Wittenberg and Wang, 2006) and frequency-dependent synaptic plasticity stimulation protocols for long-term potentiation (LTP) and long-term depression (LTD) induction (Pousinha et al., 2017) were applied to model synaptic weight changes.

A two-compartment model consists of somatic and dendritic compartments (Pinsky and Rinzel, 1994; Ferguson and Campbell, 2009).

- Somatic compartment has five ionic current channels:
  - $I_{Na,s}$ - inward Na^+ current (Pinsky and Rinzel, 1994)
  - $I_{KDR,s}$ - outward delayed rectifier K^+ current (Pinsky and Rinzel, 1994)
  - $I_{Ca,s}$ - inward Ca^{2+} current (Ferguson and Campbell, 2009)
  - $I_{KCa,s}$ - outward short-duration voltage and Ca^{2+} dependent K^+ current (Ferguson and Campbell, 2009)
  - $I_{KAHP,s}$ - outward long-duration Ca^{2+}-dependent afterhyperpolarization AHP K^+ current (Ferguson and Campbell, 2009)
  - $I_{leak,s}$ - leak current (Pinsky and Rinzel, 1994)
- Dendritic compartment has three ionic current channels:
  - $I_{Ca,d}$ - inward Ca^{2+} current (Pinsky and Rinzel, 1994)
  - $I_{KCa,d}$ - outward short-duration voltage and Ca^{2+} dependent K^+ current (Pinsky and Rinzel, 1994)
  - $I_{KAHP,d}$ - outward long-duration Ca^{2+} -dependent afterhyperpolarization AHP K^+ current (Pinsky and Rinzel, 1994)
  - $I_{leak,d}$ - leak current (Pinsky and Rinzel, 1994)

A synapse containing AMPAR and GluN2A/GluN2B-NMDAR was formed on the dendritic compartment.

A multicompartmental model of CA1 pyramidal neuron (Migliore et al., 2018) was used, and a cluster of 50 AMPARs and GluN2A/GluN2B-NMDARs containing synapses, distributed randomly on the apical dendrites in the stratum radiatum region with synaptic density of 0.8 synapses/μm of dendrite (Bezaire et al., 2016; Gasparini et al., 2004), was formed at 140 μm from the soma.

AMPAR and GluN2A/GluN2B-NMDAR currents are described in 1.2.

Stimulation protocols:

- STDP induction protocol (Wittenberg and Wang, 2006): presynaptic input was paired with a doublet of postsynaptic action potentials at 1 Hz and 5 Hz frequency, and with a single postsynaptic action potential at 5 Hz frequency. Temporal difference between pre- and postsynaptic activity was varied from -100 ms to 100 ms.
- Frequency-dependent synaptic plasticity induction protocol (Pousinha et al., 2017): presynaptic input was stimulated using a conditioning protocol that consisted of 100 pulses at 100 Hz (LTP protocol) or 100 pulses at 1 Hz (LTD protocol). Synaptic placticity outcome was measured as the change in the somatic excitatory postsynaptic potential (EPSP). To estimate the EPSP change, a presynaptic stimulus was delivered before and after the conditioning stimulation, and the resulting ratio between the maximal values of the resulting EPSPs in soma was calculated.

1.1 Two-compartment neuron model of CA1 pyramidal neuron

The model is composed of somatic and dendritic compartments connected via the coupling conductance (Pinsky and Rinzel, 1994; Ferguson and Campbell, 2009).

Membrane potential in soma V_s and dendrite V_d are defined:

$$\begin{aligned} C_m \frac{d}{dt} V_s = &- I_{leak,s}(V_s) \\ &- I_{Na,s}(V_s, h_s) \\ &- I_{KDR,s}(V_s, n_s) \\ &- I_{Ca,s}(V_{s,s_s}) \\ &- I_{KCa,s}(V_s, [Ca^{2+}]_s, c_s) \\ &- I_{KAHP,s}(V_s, q_s) \\ &+ \frac{g_c}{p}(V_d - V_s) + \frac{I_s}{p}, \end{aligned} \tag{S1}$$

$$\begin{aligned} C_m \frac{d}{dt} V_d = &- I_{leak,d}(V_d) \\ &- I_{Ca,d}(V_{d,s_d}) \\ &- I_{KC,d}(V_d, [Ca^{2+}]_d, c_d) \\ &- I_{KAHP,d}(V_{d,q_d}) \\ &+ \frac{g_c}{1-p}(V_s - V_d) - \frac{I_{syn}}{1-p}, \end{aligned} \tag{S2}$$

where C_m is the membrane capacitance per unit area, $I_{Na,s}$ is inward Na^+ current in soma, $I_{KDR,s}$ is outward delayed rectifier K^+ current in soma, $I_{Ca,s}$ is inward Ca^{2+} current in soma, $I_{KCa,s}$ is outward short-duration voltage and Ca^{2+} dependent K^+ current in soma, $I_{KAHP,s}$ is outward long-duration Ca^{2+}-dependent AHP K^+ current in soma, $I_{leak,s}$ is leak current in soma, $I_{Ca,d}$ is inward Ca^{2+} current in dendrite, $I_{KCa,d}$ is outward short-duration voltage and Ca^{2+} dependent K^+ current in dendrite, $I_{KAHP,d}$ is outward long-duration Ca^{2+}-dependent AHP K^+ current in dendrite, $I_{leak,d}$ is leak current in dendrite, I_s is the external current applied to the soma, p is the proportion of cell area taken by the soma, g_c is the coupling conductance between the somatic and dendritic compartments.

The ionic currents in Eq.S1 and Eq.S2 are described using Hodgkin-Huxley formalism.

Leak current in somatic and dendritic compartments $I_{leak,\star}(V_\star)$ are equal:

$$I_{leak,\star}(V_\star) = \hat{g}_{leak,\star}\,(V_\star - E_{leak}), \tag{S3}$$

where $\star \in \{s, d\}$ denotes somatic and dendritic compartments.

Inward Na^+ current $I_{Na,s}(V_s, h_s)$ in soma is responsible for action-potential generation and depends on the membrane potential in soma V_s, activation variable m_∞ and inactivation variable h_s:

$$I_{Na,s}(V_s, h_s) = \hat{g}_{Na,s}\, m_\infty^2\, h_s\,(V_s - E_{Na}), \tag{S4}$$

Outward delayed rectifier K^+ current $I_{KDR,s}$ in soma delays action potential generation and depends on V_s and activation variable n_s:

$$I_{KDR,s}(V_s, n_s) = \hat{g}_{KDR,s}\, n_s\,(V_s - E_K), \tag{S5}$$

Inward Ca^{2+} current $I_{Ca,\star}$ is modeled in somatic and dendritic compartments, here $\star \in \{s, d\}$. This current is sensitive to the local membrane potential $V_\star$ and activation variable $s_\star$:

$$I_{Ca,\star}(V_{\star,s_\star}) = \hat{g}_{Ca,\star}\, s_\star^2\,(V_\star - E_{Ca}), \tag{S6}$$

The activation of KCa and $KAHP$ ion channels depends on the intracellular Ca^{2+} concentration $[Ca^{2+}]_\star$.

Outward short-duration voltage and Ca^{2+} dependent K^+ current $I_{KCa,\star}$ is present in somatic and dendritic compartments and proportional to the gating variable $c_\star$ and saturating function $\lambda_\star$:

$$I_{KCa,\star}(V_\star, [Ca^{2+}]_\star, c_\star) = \hat{g}_{KCa,\star}\, c_\star\, \lambda_\star\,(V_\star - E_K), \tag{S7}$$

where $\lambda_\star$ is a function of local $[Ca^{2+}]_\star$ and is given in Eq.S11.

Outward long-duration calcium-dependent AHP potassium current $I_{KAHP,\star}$ in soma and dendrite depends on the local membrane potential $V_\star$ and a local intracellular calcium concentration $[Ca^{2+}]_\star$-dependent gating variable $q_\star$:

$$I_{KAHP,\star}(V_{\star,q_\star}) = \hat{g}_{KAHP,\star}\, q_\star\,(V_\star - E_K), \tag{S8}$$

In somatic compartment, intracellular calcium concentration $[Ca^{2+}]_s$ increases due to the $I_{Ca,s}$ current:

$$\frac{d}{dt}[Ca^{2+}]_s = -\phi \times I_{Ca,s} - \beta_{[Ca^{2+}]}[Ca^{2+}]_s \tag{S9}$$

where ϕ is the scaling constant that converts the inward calcium current to the intracellular calcium concentration $[Ca^{2+}]_s$, and $\beta_{[Ca^{2+}]}$ defines calcium decay via the calcium pump and buffering.

In dendritic compartment, NMDAR-mediated calcium current $I_{Ca,NMDA}$ (Eq.S34) contributes to the intracellular calcium concentration $[Ca^{2+}]_d$:

$$\frac{d}{dt}[Ca^{2+}]_d = -\phi \times (I_{Ca,d} + I_{Ca,NMDA}) - \beta_{[Ca^{2+}]}[Ca^{2+}]_d. \tag{S10}$$

Intracellular calcium concentration $[Ca^{2+}]_\star$ influences the saturation function $\lambda_\star$ that activates $I_{KCa,\star}$ (Eq.S7):

$$\lambda_\star = min(1, [Ca^{2+}]_\star/250). \tag{S11}$$

The gating variables h_s, n_s, $s_\star$, $c_\star$ are described:

$$\frac{d}{dt}y_\star = \frac{y_\infty(V_\star) - y_\star}{\tau_y(V_\star)}, \tag{S12}$$

where $y \in \{h, n, s, c\}$.

The gating variable $q_\star$ is equal:

$$\frac{d}{dt}q_\star = \frac{q_\infty([Ca^{2+}]_\star) - q_\star}{\tau_q([Ca^{2+}]_\star)}, \tag{S13}$$

The steady state value and time constant of the gating variables h_s, n_s, $s_\star$, $c_\star$ are defined:

$$y_\infty(V_\star) = \frac{\alpha_y(V_\star)}{\alpha_y(V_\star) + \beta_y(V_\star)} \tag{S14}$$

and

$$\tau_y(V_\star) = \frac{1}{\alpha_y(V_\star) + \beta_y(V_\star)}. \tag{S15}$$

The steady state value and time constant of the gating variable $q_\star$ are defined:

$$q_\infty([Ca^{2+}]_\star) = \frac{\alpha_q([Ca^{2+}]_\star)}{\alpha_q([Ca^{2+}]_\star) + \beta_q([Ca^{2+}]_\star)} \tag{S16}$$

and

$$\tau_q([Ca^{2+}]_\star) = \frac{1}{\alpha_q([Ca^{2+}]_\star) + \beta_q([Ca^{2+}]_\star)}. \tag{S17}$$

Rate constants α_y and β_y are defined below.

Rate constants α_m and β_m for $I_{Na,s}$ activation are equal:

$$\alpha_m(V_s) = \frac{0.32 \times (-46.9 - V_s)}{e^{(46.9 - V_s)/4} - 1}, \tag{S18}$$

$$\beta_m(V_s) = \frac{0.28 \times (V_s + 19.9)}{e^{(V_s + 19.9)/5} - 1}, \tag{S19}$$

$$m_\infty = \frac{\alpha_m(V_s)}{\alpha_m(V_s) + \beta_m(V_s)} \tag{S20}$$

Rate constants α_h and β_h for $I_{Na,s}$ inactivation are equal:

$$\alpha_h(V_s) = 0.128 \times e^{(43.0 - V_s)/18.0}, \tag{S21}$$

$$\beta_h(V_s) = \frac{4.0}{e^{(-20.0 - V_s)/5} + 1}, \tag{S22}$$

Rate constants α_n and β_n for $I_{KDR,s}$ activation are equal:

$$\alpha_n(V_s) = \frac{0.016 \times (-24.9 - V_s)}{e^{(-24.9 - V_s)/5} - 1}, \tag{S23}$$

$$\alpha_n(V_s) = 0.25 \times e^{(-1.0 - 0.025 \times V_s)/18.0}, \tag{S24}$$

Rate constants α_q and β_q for $I_{KAHP,\star}$ activation depend on $[Ca^{2+}]_\star$:

$$\alpha_q([Ca^{2+}]_\star) = min(0.00002 \times [Ca^{2+}]_\star, 0.01), \tag{S25}$$

$$\beta_q = 0.001, \tag{S26}$$

Rate constants α_c and β_c for $I_{KCa,\star}$ activation also depend on $[Ca^{2+}]_\star$:

$$\alpha_c(V_\star) = \begin{cases} 2 \times e^{(53.5 - V_\star)/27} & \text{if } V_s > -10mV \\ (e^{(V_\star + 50)/11 - (53.5 + V_\star)/27})/18.975 & \text{otherwise} \end{cases} \tag{S27}$$

$$\beta_c(V_\star) = \begin{cases} 0 & \text{if } V_s > -10mV \\ 2 \times e^{(-53.5 - V_\star)/27} - \alpha_c(V_\star) & \text{otherwise} \end{cases} \tag{S28}$$

Rate constants α_s and β_s for $I_{KCa,\star}$ are equal:

$$\alpha_s(V_\star) = \frac{1.6}{1 + e^{(-0.072 \times V_\star) - 1}}, \tag{S29}$$

$$\beta_s(V_\star) = \frac{0.02 \times (V_\star + 8.9)}{e^{(V_\star + 8.9)/5} - 1}, \tag{S30}$$

The parameters are given in Table S1.

1.2 AMPAR and NMDAR synapse

Synaptic current I_{syn} consist of AMPAR and NMDAR-mediated currents:

$$I_{syn} = I_{AMPA} + I_{NMDA}. \tag{S31}$$

The non-specific current through the AMPAR gated channel is:

$$I_{AMPA} = w(t) g_{AMPA}(t)(V_d - E_{AMPA}), \tag{S32}$$

where $w(t)$ is a synaptic weight defined by Eq. 1.

Current through NMDAR gated channel consists of sodium $I_{Na,NMDA}$ and calcium $I_{Ca,NMDA}$ currents $I_{NMDA} = I_{Na,NMDA} + I_{Ca,NMDA}$ that are expressed:

$$I_{Na,NMDA} = 0.94 \times g_{NMDA}(t)(V_d - E_{NMDA}), \tag{S33}$$

$$I_{Ca,NMDA} = 0.06 \times g_{NMDA}(t)(V_d - E_{NMDA}). \tag{S34}$$

Synaptic conductances of AMPAR and NMDAR-mediated currents were simulated using a kinetic model of postsynaptic receptors (Destexhe et al., 1994). Presynaptic activation was modeled as a brief pulse of glutamate concentration (1 mM during 1 ms) that triggered binding of the transmitter to AMPAR and NMDAR, and induced transition of receptors from closed to open states.

AMPAR synaptic conductance is described (Destexhe et al., 1994):

$$g_{AMPA} = (R_{on_{AMPA}} - R_{off_{AMPA}})\hat{g}_{AMPA}, \tag{S35}$$

where $R_{on_{AMPA}}$ and $R_{off_{AMPA}}$ are the fraction of open and closed AMPAR, $\hat{g}_{AMPA}$ is the maximal AMPAR conductance.

$R_{on_{AMPA}}$ and $R_{off_{AMPA}}$ and $R_{inf_{AMPA}}$ are equal:

$$\tau_{AMPA} \frac{d}{dt} R_{on_{AMPA}} = (R_{inf_{AMPA}} - R_{on_{AMPA}}), \tag{S36}$$

$$\frac{d}{dt} R_{off_{AMPA}} = -\beta_{AMPA} R_{off_{AMPA}}, \tag{S37}$$

and

$$R_{inf_{AMPA}} = \frac{\alpha_{AMPA}}{\alpha_{AMPA} + \beta_{AMPA}}, \tag{S38}$$

where α_{AMPA} and β_{AMPA} are forward and backward binding rates are of AMPAR. Time constant τ_{AMPA} of AMPAR activation is defined:

$$\tau_{AMPA} = \frac{1}{\alpha_{AMPA} + \beta_{AMPA}}, \tag{S39}$$

The parameters of AMPAR and NMDAR are given in Table S2.

2 SUPPLEMENTARY TABLES

Table S1. Parameters of a two-compartmental model of CA1 pyramidal neuron

Parameter	Value	Unit	Description	Ref
Parameters of the two-compartmental model of CA1 pyramidal neuron.				
$V_{leak,s}$	-65	mV	Leak reversal potential in soma	Pinsky and Rinzel (1994)
$V_{leak,d}$	-65	mV	Leak reversal potential in dendrite	Pinsky and Rinzel (1994)
C_m	3	$\mu F/cm^2$	Membrane capacitance	Pinsky and Rinzel (1994)
$\hat{g}_{leak,s}$	0.1	mV	Maximum leakage conductance in soma	Pinsky and Rinzel (1994)
$\hat{g}_{leak,d}$	0.1	$\mu F/cm^2$	Maximum leakage conductance in dendrite	Pinsky and Rinzel (1994)
$\hat{g}_{Na,s}$	30	$\mu F/cm^2$	Maximum conductance of $I_{Na,s}$ in soma	Pinsky and Rinzel (1994)
$\hat{g}_{KDR,s}$	17	$\mu F/cm^2$	Maximum conductance of $I_{KDR,s}$ in soma	Ferguson and Campbell (2009)
$\hat{g}_{Ca,s}$	6	$\mu F/cm^2$	Maximum conductance of $I_{Ca,s}$ in soma	Ferguson and Campbell (2009)
$\hat{g}_{KCa,s}$	15	$\mu F/cm^2$	Maximum conductance of $I_{KCa,s}$ in soma	Pinsky and Rinzel (1994)
$\hat{g}_{KAHP,s}$	0.8	$\mu F/cm^2$	Maximum conductance of $I_{KAHP,s}$	Ferguson and Campbell (2009)
$\hat{g}_{Ca,d}$	5	$\mu F/cm^2$	Maximum conductance of $I_{Ca,d}$	Pinsky and Rinzel (1994)
$\hat{g}_{KCa,d}$	5	$\mu F/cm^2$	Maximum conductance of $I_{KCa,d}$ in dendrite	Pinsky and Rinzel (1994)
$\hat{g}_{KAHP,d}$	0.8	$\mu F/cm^2$	Maximum conductance of $I_{KAHP,d}$ in dendrite	Pinsky and Rinzel (1994)
g_c	1.5	mS/cm^2	Coupling conductance between somatic and dendritic compartments	Pinsky and Rinzel (1994)
p	0.5	$-$	Proportion of the cell area taken by soma	Pinsky and Rinzel (1994)
E_{Na}	60	mV	Reversal potential for Na^+	Pinsky and Rinzel (1994)
E_{Ca}	80	mV	Reversal potential for Ca^{2+}	Pinsky and Rinzel (1994)
E_K	-75	mV	Reversal potential for Na^+	Pinsky and Rinzel (1994)
I_s	20	$\mu A/cm^2$	Stimulus current pulse injected in soma for STDP protocol $5ms$	adjusted

Table S2. Parameters of NMDAR and AMPAR synapses. Parameter values are presented for synapses in two-compartmental model and multicompartmental model (in parentheses, if different) of CA1 pyramidal neuron

Parameter	Value	Unit	Description	Ref
		AMPA and NMDA receptors		
α_{GluN2A}	0.5	$/ms$	Forward binding rate of AMPAR	(Destexhe et al., 1994)
β_{AMPA}	0.19	$/ms$	Backward binding rate of AMPAR	(Destexhe et al., 1994)
$\hat{g}_{AMPA}$	1 x 10^{-2} (8 x 10^{-5})	nS	Maximal AMPAR conductance	adjusted
E_{AMPA}	0	mV	AMPA reversal potential	(Destexhe et al., 1994)
E_{NMDA}	0	mV	NMDA reversal potential	(Destexhe et al., 1994)

REFERENCES

Bezaire, M. J., Raikov, I., Burk, K., Vyas, D., and Soltesz, I. (2016). Interneuronal mechanisms of hippocampal theta oscillations in a full-scale model of the rodent CA1 circuit. *eLife* 5, e18566. doi:10.7554/eLife.18566

Destexhe, A., Mainen, Z. F., and Sejnowski, T. J. (1994). Synthesis of models for excitable membranes, synaptic transmission and neuromodulation using a common kinetic formalism. *Journal of Computational Neuroscience* 1, 195–230. doi:10.1007/BF00961734

Ferguson, K. and Campbell, S. (2009). A Two Compartment Model of a CA1 Pyramidal Neuron. *The Canadian Applied Mathematics Quarterly* 17

Gasparini, S., Migliore, M., and Magee, J. C. (2004). On the Initiation and Propagation of Dendritic Spikes in CA1 Pyramidal Neurons. *Journal of Neuroscience* 24, 11046–11056. doi:10.1523/JNEUROSCI.2520-04.2004

Migliore, R., Lupascu, C. A., Bologna, L. L., Romani, A., Courcol, J.-D., Antonel, S., et al. (2018). The physiological variability of channel density in hippocampal CA1 pyramidal cells and interneurons explored using a unified data-driven modeling workflow. *PLOS Computational Biology* 14, e1006423. doi:10.1371/journal.pcbi.1006423

Pinsky, P. F. and Rinzel, J. (1994). Intrinsic and network rhythmogenesis in a reduced Traub model for CA3 neurons. *Journal of Computational Neuroscience* 1, 39–60. doi:10.1007/BF00962717

Pousinha, P. A., Mouska, X., Raymond, E. F., Gwizdek, C., Dhib, G., Poupon, G., et al. (2017). Physiological and pathophysiological control of synaptic GluN2B-NMDA receptors by the C-terminal domain of amyloid precursor protein. *eLife* 6. doi:10.7554/eLife.25659

Wittenberg, G. M. and Wang, S. S.-H. (2006). Malleability of Spike-Timing-Dependent Plasticity at the CA3-CA1 Synapse. *Journal of Neuroscience* 26, 6610–6617. doi:10.1523/JNEUROSCI.5388-05.2006
